# In vivo microscopy reveals macrophage polarization locally promotes coherent microtubule dynamics in migrating cancer cells

**DOI:** 10.1038/s41467-020-17147-y

**Published:** 2020-07-14

**Authors:** Gaurav Luthria, Ran Li, Stephanie Wang, Mark Prytyskach, Rainer H. Kohler, Douglas A. Lauffenburger, Timothy J. Mitchison, Ralph Weissleder, Miles A. Miller

**Affiliations:** 1 Center for Systems Biology, Massachusetts General Hospital Research Institute, Boston, MA 02114 USA; 2 Department of Biomedical Informatics, Harvard Medical School, Boston, MA 02115 USA; 3 Department of Radiology, Massachusetts General Hospital and Harvard Medical School, Boston, MA 02115 USA; 4 Department of Biological Engineering, Massachusetts Institute of Technology, Cambridge, MA 02181 USA; 5 Department of Systems Biology, Harvard Medical School, Boston, MA 02115 USA

**Keywords:** Cancer microenvironment, Cellular imaging, Microtubules

## Abstract

Microtubules (MTs) mediate mitosis, directional signaling, and are therapeutic targets in cancer. Yet in vivo analysis of cancer cell MT behavior within the tumor microenvironment remains challenging. Here we developed an imaging pipeline using plus-end tip tracking and intravital microscopy to quantify MT dynamics in live xenograft tumor models. Among analyzed features, cancer cells in vivo displayed higher coherent orientation of MT dynamics along their cell major axes compared with 2D in vitro cultures, and distinct from 3D collagen gel cultures. This in vivo MT phenotype was reproduced in vitro when cells were co-cultured with IL4-polarized MΦ. MΦ depletion, MT disruption, targeted kinase inhibition, and altered MΦ polarization via IL10R blockade all reduced MT coherence and/or tumor cell elongation. We show that MT coherence is a defining feature for in vivo tumor cell dynamics and migration, modulated by local signaling from pro-tumor macrophages.

## Abstract (web summary)

The regulation of microtubule (MT) dynamics in cancer cells within the tumor microenvironment is less understood. Here, the authors develop an imaging platform to examine MT dynamics in live xenograft models and show that pro-tumor macrophages modulate MT coherence and alignment to promote cancer cell migration.

## Introduction

The cytoskeleton coordinates cellular morphology, mitosis, and migration. It consists most prominently of actin filaments (F-actin) and microtubules (MTs)^1,2^, both of which are maintained through a balance of near constant polymerization and depolymerization^3^. While F-actin forms branched networks, MTs are often longer (>50 μm in some cases), straighter, and turn over more slowly (every 3–5 min)^4^. F-actin governs rapid cycles of cytoskeletal protrusion, adhesion, and contraction to move cells. However, MTs control these cycles by establishing cell polarization through directional trafficking. MT-controlled molecular asymmetry between the front and rear end of a migrating cell—and the selective allocation of focal adhesion proteins, proteases, and secretory vesicles—is critical for cellular migration^5–7^.

Persistent cellular migration is central to development, wound healing, and immune responses. Cancer cell migration drives steps of metastasis, responsible for 90% of cancer death^8^. Reports have investigated how MTs are regulated within the tumor microenvironment (TME). However, most studies have used 2D monocultures^9,10^, which do not reflect the complex environments that cancer cells encounter in vivo. MTs are typically more important for cell shape and migration in 3D cultures than on stiff 2D substrates, and 3D culture is more representative of in vivo mechanical and adhesion environments^11,12^. Cell treatment with MT stabilizing (e.g., paclitaxel) and destabilizing (e.g., nocodazole) agents results in loss of protrusions required for migration in 3D collagen gels. However, in stiff 2D cultures, these agents can minimally impact cellular migration^13,14^. Despite improvements in 3D culture, TME complexity may never be fully reproduced in vitro, considering its dynamic cellular composition. Multiple signaling pathways influence MT dynamics, including via receptor tyrosine kinases (RTKs) and G-protein coupled receptors^15,16^. Nonetheless, it is unclear how these act in the TME where multiple such pathways operate simultaneously, thus begging the question: if MT dynamics and cell migration occur so distinctly in various tissue culture models, how do MTs actually behave in vivo?

To address these issues, we developed an integrated pipeline combining in vivo confocal (intravital) microscopy^17^, automated plus-end tip tracking of individual MTs^18^, and multivariate statistics to study MT dynamics in live xenograft models of cancer. In multiple models, this approach revealed that in vivo, MT growth in cancer cells was aligned along the cell major axis and coherently with each other in the same cell, which correlated with elongated cell morphology, formation of MT-rich pseudopod-like structures, and cell migration. Intriguingly, these properties were especially enriched in cancer cells that neighbored tumor associated macrophages (TAMs). Coherent MT dynamics induced by neighboring MΦ were disrupted by drugs targeting epidermal growth factor (EGF) receptor (EGFR) on tumor cells, and MΦ polarization via interleukin 10 receptor, IL10R. Acute disruption in signaling and MT dynamics, via targeting of phosphoinositide 3-kinase (PI3K), preceded subsequent changes in gross cell shape, and MT-destabilizing vinblastine confirmed cellular elongation in vivo was MT-dependent. Overall, we present a platform for examining the in situ dynamics of MTs in live xenograft models of cancer, revealing MT coherence as a defining feature of in vivo tumor cell motility, and that pro-tumor MΦ signaling can produce such MT coherence in neighboring tumor cells.

## Results

### Imaging, detecting, and tracking in vivo EB3-mApple comets

Stable transfectants of fusion protein EB3-mApple were used to visualize plus-end MT dynamics in cancer cells. Fluorescent fusion proteins of EB3, also known as MT-associated protein RP/EB family member 3 (MAPRE3), are widely used tools to visualize MT dynamics in live cells^18^. They are relatively non-perturbing of endogenous dynamics and report activity of MT-targeting drugs in cancer^19,20^. As a model system, we used HT1080 human fibrosarcoma cells, since they have been characterized, including by intravital microscopy (IVM), for their migratory behavior, responsiveness to MT-targeting therapies, and distinct cytoskeletal characteristics in 2D vs. 3D tissue cultures^19–24^. Dorsal window chambers were implanted over subcutaneous HT1080-EB3-mApple xenografts for longitudinal imaging. To help distinguish tumor cells from each other, only a fraction expressed EB3-mApple.

**Fig. 1:**
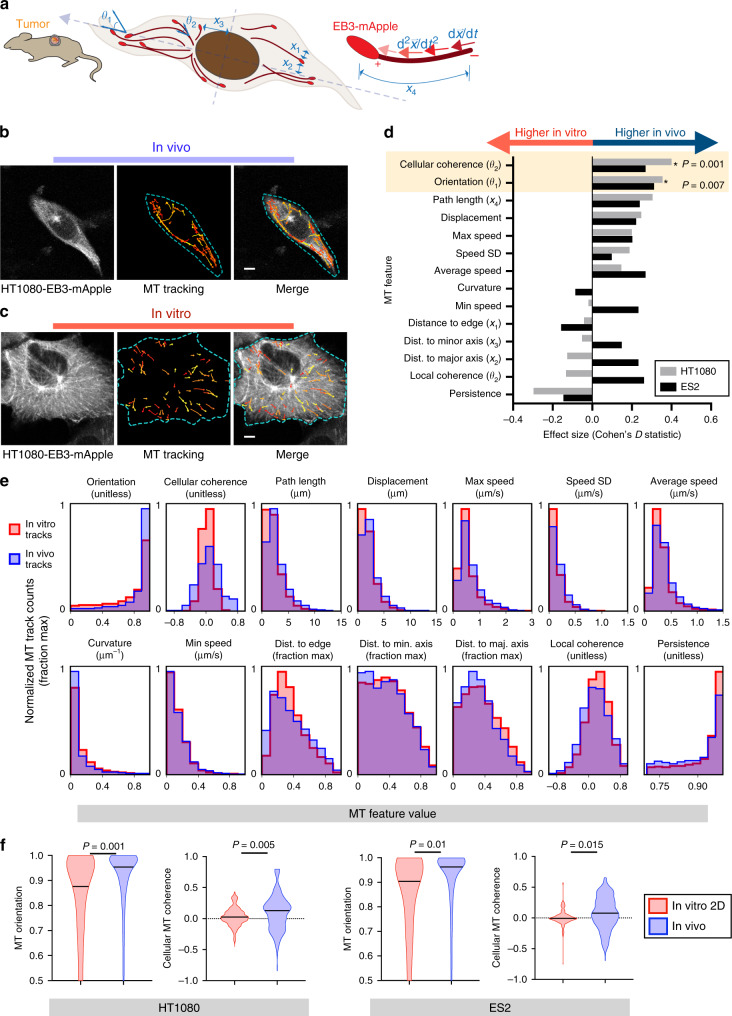
Microtubule dynamics in live xenograft tumor models. **a** MT features (blue) and others were quantified from IVM of EB3-mApple comet trajectories (red). **b** Representative time-lapse IVM of cancer cells growing in a dorsal window chamber from *nu*/*nu* host (left). MTs were tracked (center) and randomly pseudo-colored for visualization. **c** Representative in vitro time-lapse images were obtained from 2D culture using the same imaging system (left) and tracking software (center). For each MT track, the (**d**) effect size was compared between in vivo and in vitro conditions along with (**e**) the corresponding distribution plot for HT1080 MT tracks (D: *two-tailed permutation test with Benjamini–Hochberg correction). **f** Distributions of MT features showing in vivo vs. in vitro differences shared for both HT1080 and ES2 xenograft models, using the same IVM setups (*two-tailed permutation test; bar denotes median). For HT1080, a total of *n* = 8126 tracks across 73 cells and 4 tumors were analyzed (**b**–**f**). For ES2 a total of *n* = 2857 tracks across 42 cells and 5 tumors were analyzed (**d**, **f**). Scale bar = 5 μm (**b**, **c**). Source data are provided as a source data file.

IVM revealed comet-like EB3-mApple behaviors consistent with previous reports^18^ (Supplementary Fig. 1). The plusTipTracker algorithm linked comets in consecutive frames to form MT trajectories. We manually examined tracking accuracy across cells, finding a false positive rate of <5% (11/300; Supplementary Fig. 2). We computed 14 track features describing MT behavior, including MT growth speed (average, minimum, maximum, and standard deviation), cellular location (distance to the nearest cell edge, major axis, and minor axis, shown as *x*_1_, *x*_2_, and *x*_3_ respectively in Fig. 1a), orientation (*θ*_1_ in Fig. 1a), persistence, curvature, displacement, and path length (*x*_4_ in Fig. 1a). We also computed track coherence, a measure of how similar a track’s direction is to other tracks (related to *θ*_2_, Fig. 1a), at both local (within a 20 µm radius) and whole-cell levels (Supplementary Fig. 3). For instance, a cellular coherence value of 1.0 means all MT tracks move in the same direction and suggests asymmetric polarization, while symmetrically radiating MT tracks from the cell center would have a cellular coherence of 0.0. Because EB3 binds to only growing MT ends, we did not capture MT shrinkage and pausing^25^. Features were normalized to be independent of cell size.

To gauge the impact of EB3-mApple itself on observed MT dynamics, we measured cell-by-cell correlations with EB3-mApple expression, finding no significance for the majority of track features (12/14). As exceptions, displacement and path length somewhat correlated (*R*^2^ < 0.25), explainable by the technical ability to image brighter MT tracks over longer time periods, and no correlation was found after correcting for track duration (Supplementary Fig. 2C). Genomic alterations of *MAPRE3* (encoding EB3) and its expression by RNA-seq did not correlate with overall survival outcomes of cancer patients across The Cancer Genome Atlas (Supplementary Fig. 2C), suggesting that EB3 itself is not a major driver of disease progression. These analyses thus support the use of EB3 as a relatively non-perturbative tool for MT imaging.

### MT dynamics profiling reveals enhanced alignment in vivo

We quantified MT differences between cells growing in vivo compared to in vitro by performing matched analysis of the same HT1080-EB3-mApple cell line cultured on standard 2D tissue culture plastic. We also examined the ES2 human ovarian cancer (OVCA) cell line as a second model (Fig. 1, Supplementary Fig. 4). In HT1080, the average MT growth rate of in vitro tracks (0.35 ± 0.15 µm s^−1^ s.d.) and in vivo tracks (0.38 ± 0.18 µm s^−1^ s.d.) were relatively consistent with prior studies in other cell types in vitro (pig kidney LLC-PK1 cells: 0.30 ± 0.13 µm s^−1^, chinese hamster ovary CHO cells: 0.27 ± 0.11 µm s^−1^ and human keratinocyte HaCaT cells: 0.31 ± 0.12 µm s^−1^)^18,26^. There was no consistent difference in relative intracellular location of pre-filtered MT tracks in HT1080: distances from the cell edge, major axis, and minor axis revealed that the majority of MTs were closer to the cell center both in vivo and in vitro. However, in ES2, in vivo tracks were somewhat faster and further from the cell edge (Supplementary Fig. 4). These observations are consistent with known MT origination from microtubule organizing centers adjacent to the cell nucleus, and less stable and slower moving MTs at the cell periphery^27^.

Compared to all other features, the orientation and coherence of MT tracks showed the greatest consistent increase for cells grown in vivo vs. in vitro. Orientation was computed from the angle between the directional vector of the MT track, and the directional vector of the corresponding cell major axis (Fig. 1a, θ_1_). A cosine transformation caused tracks parallel to the cell major axis to have high orientation. Nearly twice as many HT1080 MT tracks were angled off of the cell’s major axis by >45^°^ (orientation <0.71) in vitro compared to in vivo (32.6 ± 0.60% vs. 16.7 ± 0.79% s.e.m., respectively), meaning MT tracks were more aligned with the cell’s major axis when cells were grown in vivo. As a related measurement, the MT coherence quantified how similarly a MT track was oriented to nearby tracks within a specified distance. A positive coherence value indicated that the track was traveling parallel to nearby tracks, while a large negative value indicated that the track was traveling antiparallel. Coherence was measured for each track at the local (within 20 µm from the track) and cellular level (across all tracks of a cell). Locally, in vivo and in vitro MT tracks displayed comparable levels of MT coherence. However, when analyzing all tracks across the whole cell, MT tracks were more aligned and coherent in vivo. In vivo HT1080 MT tracks exhibited a 3.2-fold increase in mean cellular coherence compared to tracks from cells growing in vitro (0.10 ± 0.006 vs. 0.03 ± 0.002 s.e.m., respectively). Cells were on average more elongated in vivo, which can impact MT behaviors. However, even when comparing similarly shaped in vivo vs. in vitro cells, MT orientation and coherence were still enhanced in vivo (Supplementary Fig. 5).

Differences between in vivo vs. in vitro conditions are a function of biological changes and technical biases. In subsequent imaging, we focused on the former and minimized the latter through comparisons across matched conditions. We tested (a) whether image quality could impact conclusions by creating artificially noisy images (Supplementary Fig. 7), (b) if tracking parameters were sensitive to image differences by recomputing statistics on different parameter combinations (Supplementary Fig. 8), and (c) whether individual cells were driving differences by permuting cell labels to compute significance (Supplementary Figs. 6 and 7). (d) Excluded cells capturing <80% of a cell body within the field of view were also excluded from the main analyses, and we further evaluated if their inclusion altered results (Supplementary Figs. 6 and 7). Overall, cells consistently exhibited greater MT alignment in vivo compared to 2D tissue culture plastic, in a manner robust to cell-to-cell batch effects, variation in image quality, and variation in tracking parameters.

### 3D collagen gel cell cultures display distinct MT dynamics

**Fig. 2:**
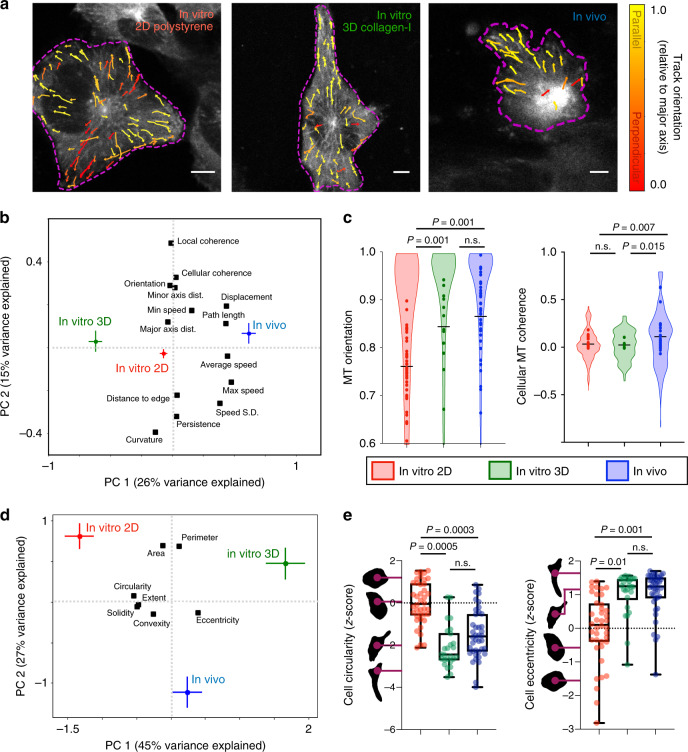
Coherent in vivo MT dynamics are not fully reproduced by 3D collagen in vitro cultures. **a** Representative confocal images of HT1080-EB3-mApple cells grown as indicated, with tracks pseudo-colored according to MT orientation along the major length axis of the cell (scale bar = 5 μm; *n* = 85 cells across *n* ≥ 2 in vitro replicates and *n* = 4 tumors). **b** MT features were analyzed by principal components analysis (PCA), shown according to average condition score (±s.e.m.) and variable loadings (black squares; *n* = 5794 tracks, middle 95% for all features). **c** Violin plots show single-track distributions for orientation and cellular coherence (bar denotes median, cell averages overlaid as individual data points; *two-tailed permutation test; *n* = 9448 total tracks from 85 cells). **d** PCA captures covariation between cellular shape features (loadings, black) across imaging conditions (scores ±s.e.m.; *n* = 106 cells). **e** Single-cell distributions of shape features, matching **d** (*two-tailed *t* test; *n* = 106 cells; box plot bars represent the minimum, 25%tile, median, 75%tile, and maximum values*)*. Source data are provided as a source data file.

MT dynamics can differ in cells growing in 3D hydrogel materials rather than on stiff 2D surfaces. Thus, we examined if the MT dynamics of HT1080 tumor cells observed in vivo could be recapitulated in soft 3D cultures. HT1080-EB3-mApple cells were grown in 3D collagen I hydrogel, since collagen I is a major TME structural component^28^ shown to influence cell migration and metastasis^21,29,30^. We used the same imaging pipeline to track MTs within the 3D-cultured cells (Fig. 2a, Supplementary Figs. 9 and 10).

We used principal components analysis (PCA) as a dimensionality reduction method to systematically compare how the pattern of co-correlated MT features changed under 2D and 3D culture conditions relative to in vivo phenotypes. 3D-cultured cells behaved distinctly from cells grown in vivo, indicated by divergent PCA scores from the centrally positioned 2D culture (Fig. 2b). Although MT orientation increased in 3D culture (Fig. 2c), and therefore phenocopied in vivo dynamics, 5/14 features did not match in vivo behavior (Supplementary Fig. 10), including cellular MT coherence (Fig. 2c). Thus, the in vitro 3D track distribution failed to fully mimic the in vivo phenotype.

We next examined whether increased in vivo MT coherence could be explained by more elongated in vivo cell shape. We again performed PCA, but using features of gross cytoskeletal shape as variables (Fig. 2d). The first and second principal components (PC1, PC2) broadly captured cell elongation and cell size, respectively. Although more detailed and/or volumetric analysis could reveal higher-order distinctions, both in vivo and 3D-cultured cells exhibited a positive PC1 shift compared to 2D-cultured cells, characterized by decreasing cell circularity and increased elongation (Fig. 2e). Thus distinct in vivo MT dynamics could not be entirely explained by differences in cell elongation.

### TAMs regulate MT alignment in neighboring tumor cells

MTs establish directional cell polarity and juxtacrine signaling with neighboring cells^5–7^. We hypothesized that local intercellular interactions could contribute to the observed coherence of MTs in vivo. In addition to cancer cells, the TME is typically rich in other cell populations, including TAMs. TAMs are among the most abundant leukocytes in the HT1080 xenograft model^31^ and in a large fraction of patient tumors^32^. TAM accumulation has been associated with disease progression, angiogenesis, and metastasis^33,34^. Using the same tumor xenograft model, we have observed by histology, flow-cytometry, and IVM an overall ratio of roughly 1:4 in the relative content of TAMs:HT1080 tumor cells, although this varies across tumor regions^24,35^. These observations motivated us to ask whether TAMs could be contributing to the coherent MT behavior of tumor cells found in vivo.

**Fig. 3:**
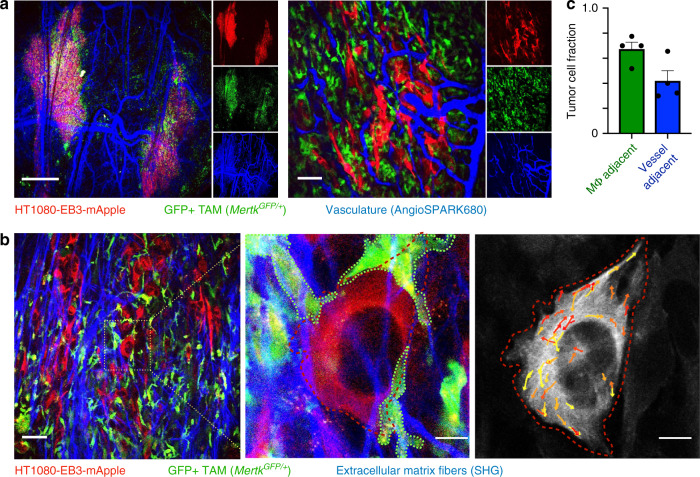
TAMs frequently neighbor tumor cells near vasculature and fibrillar extracellular matrix. **a**, **b** Representative IVM of HT1080 tumors grown subcutaneously in the dorsal window chamber of *Mertk*^*GFP/+*^ NOD.SCID mice at low (left) and high (right) magnifications. **a** A long-circulating fluorescent NP, angiospark-680, and **b** 2-photon microscopy of second harmonic generation, SHG show tumor vasculature and fibrillar extracellular matrix, respectively. At bottom right, EB3 tracks are shown randomly pseudo-colored for visualization. Scale bars are 1 mm (**a**, right), 50 μm (**a**, **b**, left), and 10 μm (**b**, right). **c** Corresponding to images as in (**a**), tumor cells were scored based on nearest proximity (<5 μm) to GFP+ TAMs or AngioSPARK+ vasculature (data are means ± s.e.m.). For all, *n* = 80 total cells and *n* = 4 tumors overlaid as individual data points in (**c**). Source data are provided as a source data file.

Multicolor IVM co-imaged MT tracks and neighboring TAMs using a genetically engineered reporter mouse model with knock-in expression of GFP in place of one functional copy of Mertk (*NOD.SCID Mertk*^*GFP/+*^), which has been validated by imaging and flow-cytometry experiments for its high and selective expression in TAMs in this xenograft model^35,36^. Mertk heterozygosity exerts minor^37^ and in some cases undetectable^38^ impacts on disease progression compared to *Mertk*^*−/*−^ phenotypes. IVM revealed that TAMs frequently neighbored or even wrapped around MT-rich tumor cell protrusions, including near vessels and fibers of collagen-rich extracellular matrix (Fig. 3).

**Fig. 4:**
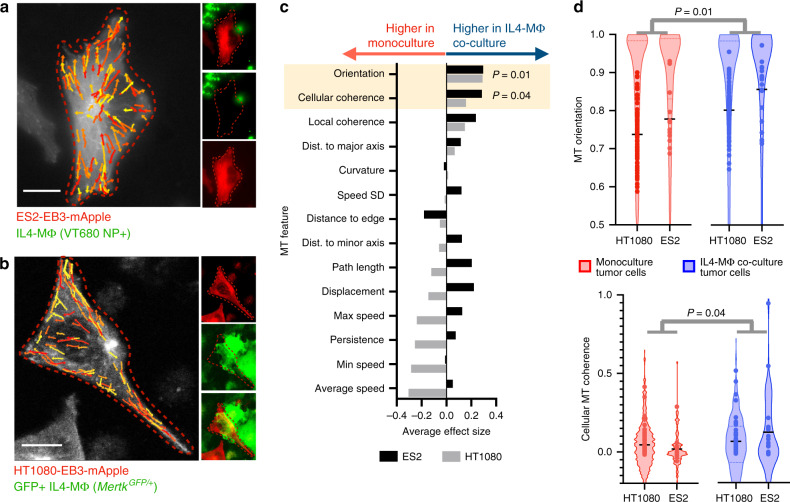
IL4-polarized MΦ promote coherent MT alignment in neighboring tumor cells. **a**, **b** Representative fluorescence microscopy after 24 h of IL4-MΦ co-culture with EB3 tracks randomly pseudo-colored for visualization (scale bar = 10 μm). **c** Corresponding to **a**, **b**, the effect size for imaged MT features was compared between monoculture and 24 h MΦ co-culture (average of Cohen’s D effect size between batches). **d** From **c**, track distributions for the top two increased MT features, altered in both cell lines with co-culture, are shown with cell averages overlaid as individual data points (bar denotes median). All *p* values were computed using a two-tailed permutation test with BH correction. For HT1080 cells, *n* = 22,371 tracks across *n* = 164 total cells were analyzed, and for ES2 cells *n* = 1424 tracks across *n* = 33 total cells were analyzed. Source data are provided as a source data file.

To test whether TAMs influence MT coherence, we analyzed EB3 MT tracks in HT1080 and ES2 cells grown ±MΦ co-culture (Fig. 4a, b). Bone-marrow-derived MΦ were differentiated by macrophage colony-stimulating factor (MCSF) and polarized with interleukin-4 to produce M2-like MΦ (referred to here as IL4-MΦ), resembling tumor-promoting TAM phenotypes^34^. After IL4-MΦ co-culturing, we imaged tumor cells in contact with MΦ and quantified their EB3 tracks. Only two MT features showed consistent changes in co-culture vs. monoculture, across both cell lines: MT coherence and orientation (Fig. 4c, d; Supplementary Figs. 11 and 12), which also differed in the in vivo vs. in vitro comparison (Fig. 1). This generalized to IL4-MΦ derived from the RAW264.7 MΦ cell line (HT1080 in Fig. 4d), thus showing MΦ influence on tumor cell MT dynamics in 2 cancer cell lines and 2 MΦ models.

### MΦ polarization impacts MT dynamics of adjacent tumor cells

**Fig. 5:**
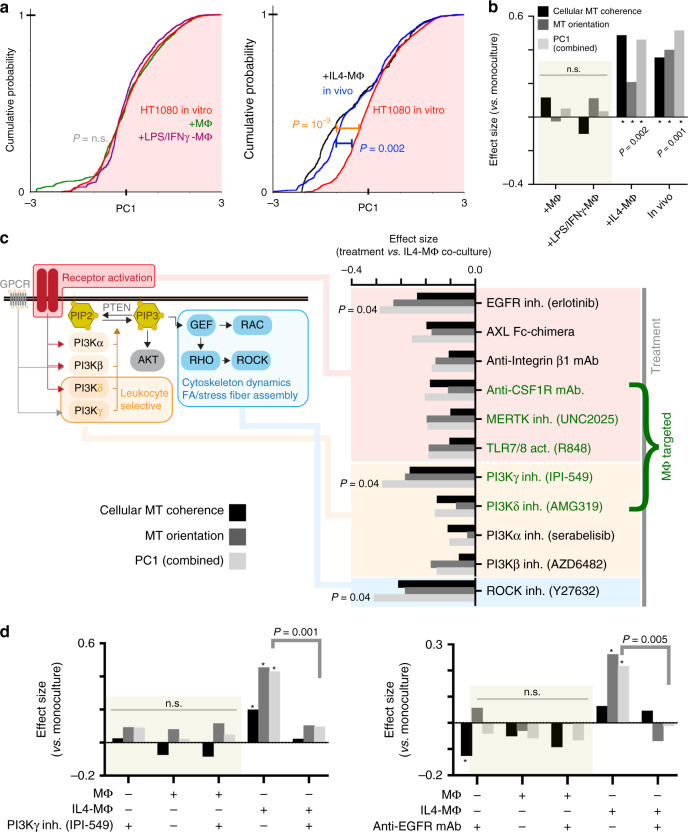
Disruption of MΦ-induced MT coherence by targeted inhibition of cell signaling. **a** HT1080 MT cellular coherence and orientation were combined into a principal component (PC) for each MT track, and the cumulative distribution function of PC scores was calculated (*two-tailed permutation test; total *n* = 10,968 tracks from *n* = 118 total cells). **b** Effect sizes were calculated from results shown in (**a**) (*two-tailed permutation test using PC scores). **c** ES2 MT dynamics were imaged after 24 h co-culture followed by 2–4 h of drug treatment, and effect sizes were calculated as in (**b**), however, here in comparison to IL4-MΦ co-culture (*two-tailed permutation test on PC scores with BH multiple hypothesis correction, fdr = 0.05; *n* = 29,591 tracks from *n* = 151 total cells). **d** HT1080 MT dynamics were imaged after 24 h co-culture followed by 2–4 h. of drug treatment, and effect sizes were calculated as in (b) (*two-tailed permutation test on PC scores; IPI-549: *n* = 13,380 tracks from *n* = 80 total cells; anti-EGFR mAb: *n* = 16,427 tracks from *n* = 112 total cells). Source data are provided as a source data file.

Although TAMs frequently exhibit tumor-promoting phenotypes, in reality they can exist across a spectrum of MΦ polarization states. We therefore examined the effect of MΦ polarization on MT dynamics in co-cultured tumor cells. MCSF-differentiated MΦ modeled what past literature has described as a M0-like phenotype, referred to here as simply MΦ^39^. We further polarized MΦ either with IL-4 (described above) or with lipopolysaccharide and interferon gamma to produce M1-like MΦ^40^, referred to here as LPS/IFNγ-MΦ. We imaged MΦ-adjacent tumor cells in each co-culture and quantified the MT features (Supplementary Figs. 11 and 12), ultimately focusing on MT coherence and orientation based on the above results. Since these two features relate to one another, yet are highly variable, we used PCA and a single principal component (PC) to capture distributions of both features simultaneously. The comparison in PC distributions revealed that only in vivo and IL4-MΦ co-culture tracks displayed higher MT coherence and orientation compared to monoculture (Fig. 5a). Pairwise differences in PC distributions (Supplementary Fig. 11) and statistical effect size (Fig. 5b) indicated IL4-MΦ co-culture tracks were most similar among all conditions to the in vivo tracks. These data suggest that TAM effects on MT dynamics depend on the underlying TAM polarization state.

### Kinase signaling disruption blocks MΦ-mediated MT coherence

TAMs and tumor cells signal bidirectionally through multiple pathways. For instance, reports show TAM-produced ligands such as EGF signal to EGFR on cancer cells to promote cell migration^41,42^. In turn, tumor cell-produced ligands such as colony-stimulating factor 1 (CSF1) signal to CSF1R on TAMs to promote recruitment and M2-like polarization^41,43^. Downstream PI3K signaling is implicated in such signaling and MT dynamics^44,45^, and in TAMs, reports show PI3K inhibition (particularly of isoform p110γ) reprograms cells toward M1-like polarization^46^.

Given this evidence, we hypothesized that receptors and PI3K govern MΦ-mediated MT polarization in tumor cells. We tested drugs and antibodies targeting (1) tumor-expressed receptors, including the RTKs EGFR and AXL, and integrin β1 (heterodimerizing with α-integrins to bind ECM); (2) TAM-expressed receptors influencing TAM polarization, including toll-like receptor 7/8 (TLR7/8) and the RTKs MERTK and CSF1R; (3) PI3K isoforms including leukocyte selective p110δ and p110γ; and (4) Rho-associated protein kinase (ROCK) as a representative effector of Rho-family GTPases. ES2 co-cultured with IL4-MΦ were treated for 2–4 h, and imaged MT features revealed that all treatments qualitatively decreased MT coherence and orientation, although only treatment with inhibitors targeting ROCK, PI3Kγ, and EGFR elicited significant effects (Fig. 5c; Supplementary Fig. 13). In support, analogous experiments in HT1080 also showed effects from targeting EGFR and PI3Kγ, under IL4-MΦ but not other co-culture or monoculture conditions (Fig. 5d; Supplementary Fig. 14). These data thus suggest that coherent MT dynamics depend on context-dependent signaling activity in both MΦ and cancer cells.

### MΦ contact promotes pseudopod-like extensions in tumor cells

**Fig. 6:**
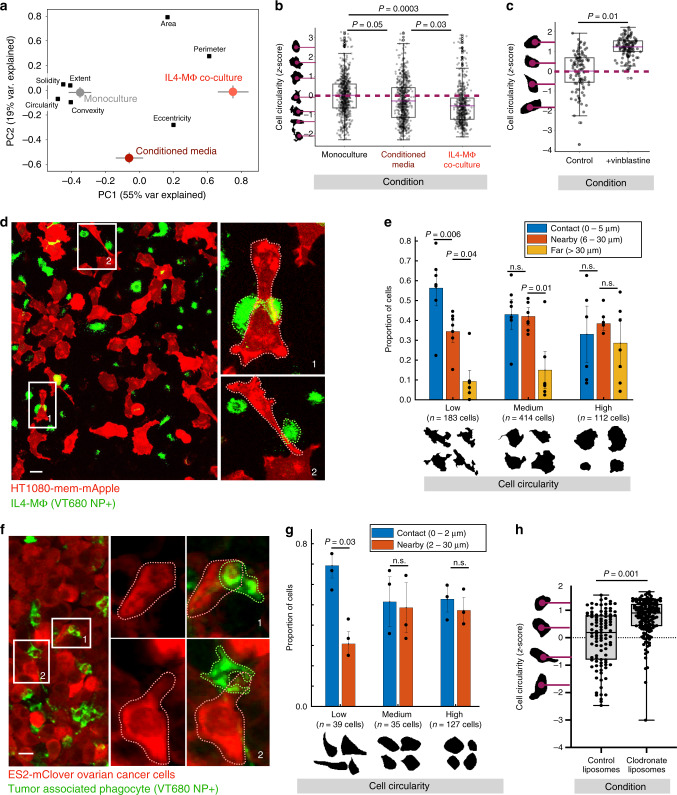
MΦ promote pseudopod-like extensions in neighboring tumor cells. **a**, **b** HT1080-mem-mApple cells were imaged after being cultured for 24 h in three different conditions. **a** PCA captures cell shape feature loadings (black) and scores for cells under these conditions (mean PC score ± s.e.m). **b** Single-cell circularity measurements, which exhibited the most negative PC1 loading in (**a**), were directly compared (*two-tailed *t* test; *n* = 1906 total cells across 23 replicate images; box plot as 25%tile, median, 75%tile with outliers outside 1.5*IQR). **c** HT1080-mem-mApple tumor-bearing nu/nu mice were treated with 6 mg kg^−1^ vinblastine or vehicle, and confocally imaged 24 h later (*two-tailed Mann-Whitney U test; *n* = 225 total cells, *n* = 4 tumors per group; box plots as in b). **d** Representative co-culture imaging, highlighting instances at right of co-localized MΦ and tumor cell protrusion (scale bar = 50 μm). **e** Cancer cell circularity from (d) was measured according to MΦ proximity (*chi-squared test; *n* = 709 total cells across *n* = 7 image replicates denoted by points; mean ± s.e.m.). **f** Representative confocal microscopy of disseminated intraperitoneal tumors, highlighting TAM-adjacent elongated tumor cells on right (scale bar = 20 μm). **g** As in (**f**), ES2 circularity was measured as a function of TAM proximity (*chi-squared test; *n* = 201 total cells across *n* = 3 tumors denoted by points; mean ± s.e.m.). **h** Excised ES2 tumors were confocally imaged for cell circularity, following treatment with PBS or clodronate liposomes (*two-tailed *t* test; *n* = 261 cells across *n* = 13 tumors per group; box plot as minimum, 25%tile, median, 75%tile, and maximum). Source data are provided as a source data file.

Although MT dynamics may outpace the kinetics of bulk change in cell shape and migration (discussed further below), under more equilibrated conditions MT structure and cell shape can correlate with one another^10^. Because IL4-MΦ enhanced MT orientation in tumor cells, we hypothesized that co-cultured IL4-MΦ would also influence tumor cell shape. To determine whether such impacts depended on spatial MΦ/tumor cell proximity, HT1080 were also grown in conditioned media that had been incubated with IL4-MΦ for 24 h and then transferred to HT1080 for 18 h. We used PCA to interpret coordinated changes in shape features (rather than MT dynamics as above). HT1080 co-cultured directly with IL4-MΦ shifted toward elongated, less circular shapes, and conditioned media elicited lower magnitude effects (Fig. 6a). The circularity shape feature exhibited the most negative loading on the first PC (Fig. 6a), substantially decreased with co-culture (Fig. 6b), and therefore was used as a representative metric in subsequent analyses. Overall, MΦ-enhanced MT coherence in tumor cells was matched with correspondingly decreased tumor cell circularity.

Although care must be taken in interpreting shape from confocal images with narrow focal planes, we nonetheless found correlation between cell shape and MT dynamics, even within heterogeneous populations of cells imaged under the same in vitro or in vivo conditions (Supplementary Fig. 15). To directly test the dependence of elongated cell shape on MT structure, we treated mice-bearing HT1080 tumors with 6 mg kg^−1^ MT-destabilizing drug vinblastine^47^. Twenty-four hours following treatment, tumors were confocally imaged to quantify cell shape from a membrane-tagged fluorescent protein (mem-mApple). Cells with intact non-fragmented nuclear morphology in vinblastine-treated tumors exhibited increased circularity (Fig. 6c) compared to cells from untreated tumors. Taken together, MT dynamics correlated with elongated cell shape, and MT polymerization was required for such elongation in vivo.

We next analyzed the spatial dependence by which MΦ could impact tumor cell shape. Although HT1080 and IL4-MΦ co-cultures were well-mixed, stochastic distributions created diversity in the distance between tumor cells and their closest neighboring MΦ (Fig. 6d). We therefore asked whether spatial MΦ proximity correlated with tumor cell circularity on a cell-by-cell basis, using an automated nearest-neighbor analysis. Segmented tumor cell circularity was computed and algorithmically binned into one of three groups (low, medium, and high; see representative cell masks in Fig. 6e). Next, we queried the proportion of cells in each group in contact (<5 µm), nearby (6–30 µm), or far (>30 µm) from the nearest MΦ. This revealed enrichment in cell elongation among cells in close proximity with IL4-MΦ: tumor cells with low circularity were five times more likely to be in contact with, rather than far from MΦ, while tumor cells with high circularity showed no such bias (Fig. 6e).

Given the in vitro evidence showing local effects of MΦ on tumor cells, we used an orthotopic mouse model of disseminated OVCA to study whether similar spatially dependent interactions could be observed in a relevant in vivo model of disease. We imaged disseminated tumors that formed roughly 1 week following intraperitoneal (i.p.) injection of ES2 cells. Upon terminal dissection of tumor-bearing organs (omentum, liver, ovary, and peritoneal wall), ES2 cells expressing cytoplasmic GFP were confocally imaged.

In this experiment, MΦ were imaged using a fluorescent polyglucose-based nanoparticle (NP) recently demonstrated to accumulate with >90% selectivity in MΦ in multiple mouse models of cancer^48^ (Fig. 6f). ES2 cells growing in intraperitoneal metastases were on average more circular than HT1080 growing on tissue culture plastic and ES2 cells growing in subcutaneous xenografts (Supplementary Fig. 16). Shape-based stratification binning was correspondingly adjusted. For each circularity bin, we queried the proportion of cells that were within 2 μm of the nearest NP + MΦ. Similar to the in vitro HT1080 experiment, elongated tumor cells exhibited a clear bias for spatial proximity to MΦ in ES2 tumors (Fig. 6g). Thus, analyses across two distinct models demonstrated that cancer cells nearby MΦ displayed a relatively more elongated shape.

To more directly test the impact of MΦ on tumor cell shape, we depleted MΦ within intraperitoneal ES2 tumors using i.p. administration of clodronate liposomes (clod-lip)^49^ (Supplementary Fig. 16), and found increased ES2 circularity (Fig. 6h). These results confirm that TAMs promote elongation in neighboring tumor cells, and furthermore show that TAMs can be manipulated to impact tumor cell morphology.

### MΦ associate with protrusions in migrating tumor cells

**Fig. 7:**
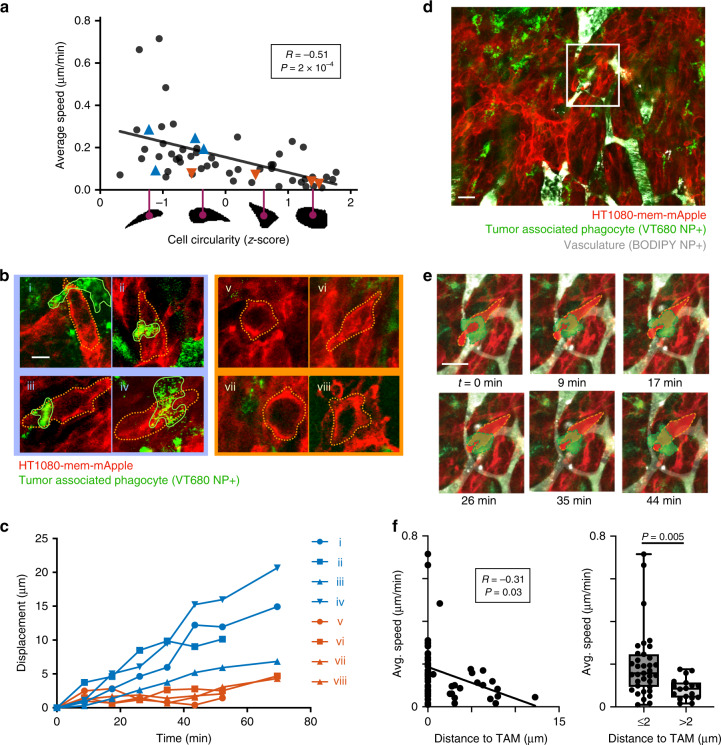
TAMs associate with pseudopod-like protrusions in migrating cancer cells. Time-lapse IVM tracked migration of individual HT1080-mem-mApple cells in tumors within the dorsal window chamber model. **a** Migration speed was correlated with cell circularity (data points are *n* = 50 individual cells across *n* = 4 tumors; *two-tailed *f* test on cell level data). Blue and red correspond to cancer cells associated with TAMs or not, respectively, in (**b**–**c**). Representative tumor cells associated with TAMs (i–iv) or not (v–viii) are shown (**b**; scale bar = 10 μm) with corresponding migration (**c**). Example TAM-associated, migrating HT1080 cell is highlighted from a 20× field of view (d; scale bar = 20 μm) and corresponding time-lapse (e; scale bar = 20 μm). Cells have been shaded for visualization. **f** Migration speeds of individual HT1080 cells were compared with their nearest distance to TAMs, shown fit to a regression (left; Pearson’s coefficient and two-tailed *t* test) and binned by distance (right; *two-tailed *t* test on cell level data; box plot as min, 25%tile, median, 75%tile, and max; **e**, **f** *n* = 55 cells across *n* = 4 tumors). Source data are provided as a source data file.

We next examined whether MΦ-induced changes in MT coherence and cellular elongation corresponded to enhanced cancer cell migration. Because of known morphological differences between motile and non-motile cells^10^, we hypothesized that tumor cells with a more circular morphology had slower migration rates than elongated cells. We used roughly 2 h time-lapse IVM (as in Fig. 1; see ref. ^24^) of HT1080 xenografts to measure cell migration by tracking individual cell centroid movements. The migration rate for all cells was 0.15 ± 0.14 μm min^−1^ (mean ± s.d.). As predicted, cell circularity correlated negatively with cell migration, and all cells with migration >0.25 µm min^−1^ had circularity ≤0.4 (Fig. 7a).

In addition, we tracked tumor phagocytes including TAMs by simultaneously imaging a fluorescently tagged NP (Fig. 7b)^24^. Examples show phagocytic myeloid cells associating with and wrapping around protrusions of migrating cancer cells (Fig. 7b, c), which correlated with greater cellular elongation and more rapid migration compared to non-TAM-associated cancer cells within the same tumor (Fig. 7d–f; Supplementary Fig. 18). These data (Fig. 7e) show that TAMs do not always co-migrate with tumor cells. Relatedly, MT dynamics were only mildly correlated with subcellular TAM positions relative to tumor cells (Supplementary Fig. 18), such that TAMs were not always located at the leading tip of elongated tumor cells. These high-resolution data suggest that TAMs may not directly lead, but nonetheless interact with and likely guide cancer cells in a localized manner.

### Acute changes in MT dynamics precede cell shape changes

**Fig. 8:**
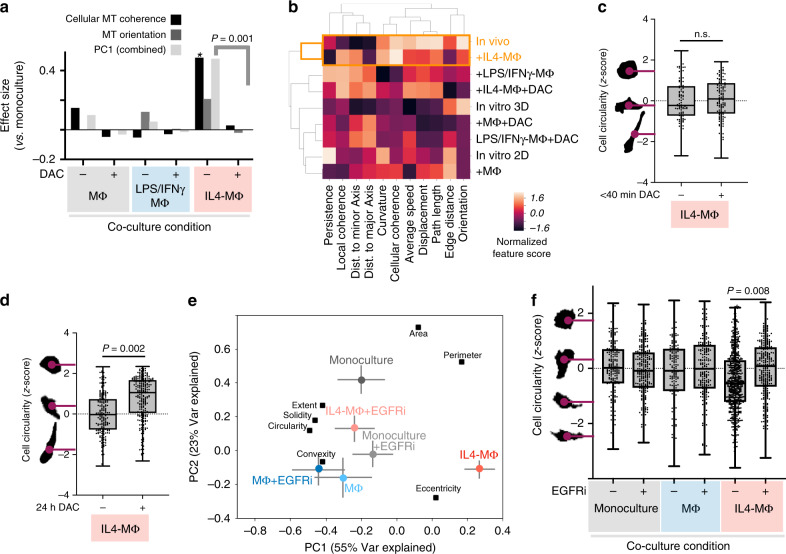
Drug-induced changes in MT dynamics precede changes in overall cell shape. **a** After 24 h co-culture, HT1080 MT dynamics were imaged from 5–40 min following 10 μM mTOR/pan-PI3K inhibitor dactolisib (DAC), and effect sizes were calculated as in Fig. 5a, b, comparing to untreated monoculture (*two-tailed permutation test; *n* = 12,524 tracks from *n* = 109 total cells). Corresponding to **a**, MT tracks were clustered according to average values (**b**), and tumor cell shape quantified following DAC at <40 min (**c**) or 24 h (**d**) post treatment (*two-tailed Mann–Whitney *U* test; **c** *n* = 223 cells, **d** *n* = 413 cells). **e**–**f** HT1080 were treated for 24 h with anti-EGFR mAb (EGFRi), quantified for cell shape, and analyzed by PCA (**e** *n* = 1594 total cells; mean ± s.e.m.) and for cell circularity (*two-tailed *t* test; *n* = 1678 cells; bar denotes median). All box plots are min, 25%tile, median, 75%tile, and max. Source data are provided as a source data file.

Given their correlation under relatively equilibrated conditions, are MT dynamics simply a reflection of cell shape? We hypothesized that acute (<2 h) changes in MT coherence would precede or predict subsequent changes in overall cell shape. As a proof-of-principle, we broadly targeted signaling using the first PI3K inhibitor to enter clinical trials, dactolisib (BEZ-235), which dually inhibits mTOR and pan-class PI3K signaling. EB3 dynamics were immediately imaged beginning 5 min post-treatment using HT1080 co-cultures. Dactolisib largely reversed the coherent MT phenotype induced by IL4-MΦ co-culture (Fig. 8a, b). At early imaged time-points there was no visual evidence for cell death or apoptosis, nor did cellular circularity substantially change (Fig. 8c). However, 24 h post treatment, co-cultured cells increased circularity (Fig. 8d). This suggests that MΦ-enhanced MT coherence depends on mTOR/PI3K-signaling pathways in a manner acutely decoupled from broader changes in cell shape, but in a more interrelated manner at longer timescales. To test effects of a selective perturbation, we used PCA to assess cell shape under co-culture conditions ±24 h anti-EGFR mAb treatment. IL4-MΦ co-culture elicited the strongest phenotype, reflected by a shift toward a positive PC1 score (Fig. 8e) and correspondingly reduced cell circularity (Fig. 8f), which was largely reversed upon EGFR inhibition. These results mirror the short-term impact of anti-EGFR on MT dynamics (Fig. 5d), and further suggest linkage between acute MT dynamics and subsequent cell shape.

### Targeting of IL10R regulates MΦ polarization and MT coherence

**Fig. 9:**
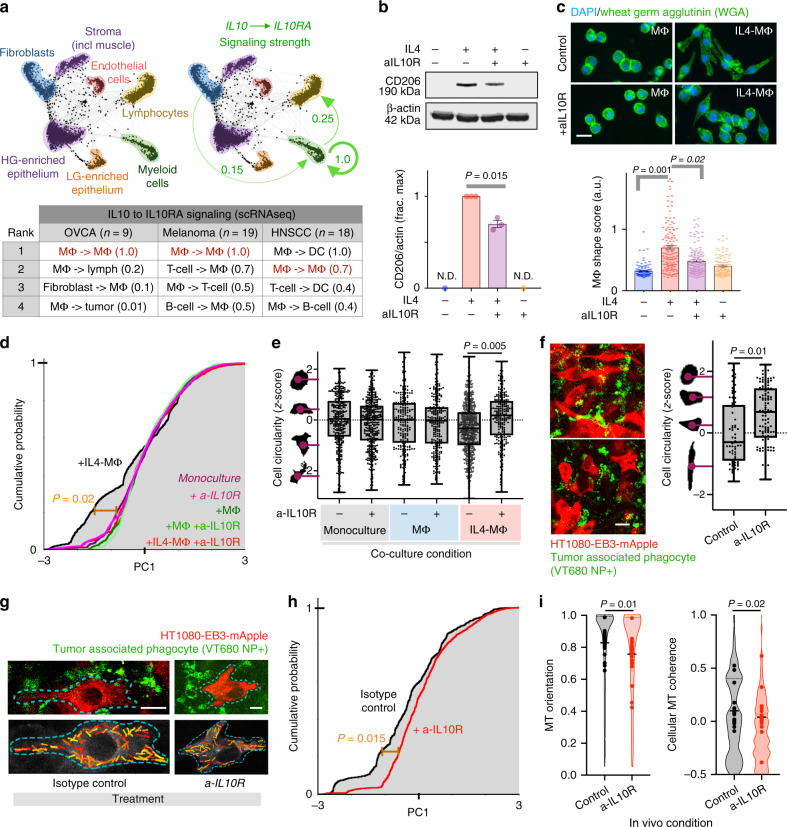
Anti-IL10R antibody shifts MΦ polarization and reduces tumor cell MT coherence. **a** SPRING visualizes scRNAseq data by cell-type from 9 patient biopsies, most either high-grade (HG) or low-grade (LG) serous OVCA (*n* > 2,900 total cells). Arrows and table report top values for [IL10xIL10RA] (fraction max) from published patient cohorts^68–70^. **b** CD206 western blot and densitometry 24 h following aIL10R in RAW264.7 (*two-tailed *t* test; *n* = 3; mean ± s.e.m.). **c** IL4-induced elongation in MΦ shape (calculated from combined shape features; scale bar = 10 μm), was monitored 24 h post-aIL10R (*two-tailed *t* test; *n* = 100 RAW264.7 cells per group with 4 groups; means ± s.e.m.). HT1080 MT dynamics (**d**) and cell circularity (**e**) were quantified as in Fig. 5, 4 h following treatment with aIL10R (**d**: *two-tailed permutation test; *n* = 21,218 tracks from 99 total cells; **e** *two-tailed *t* test; *n* = 1,964 total cells). **f** Representative IVM of TAM-proximal HT1080 following 48 h treatment with aIL10R (bottom left) or the isotype control (top left), in dorsal window chamber of nu/nu subjects, and corresponding HT1080 circularity (right; *two-tailed *t* test on cell data; *n* = 177 total cells; scale bar = 25 μm). **g**–**i** MT dynamics were captured from (**f**) and analyzed as in (**d**) (*two-tailed permutation test; *n* = 2454 tracks and 51 total cells across *n* = 8 tumors; bars denote mean; scale bar, 10 μm). All box plots are min, 25%tile, median, 75%tile, and max. Source data are provided as a source data file.

We hypothesized that in vivo MT coherence depended on the polarization state of neighboring TAMs, and therefore imaged MT dynamics in tumor-bearing subjects systemically treated with either an antibody blocking murine IL10R ligand engagement (anti-IL10R mAb, referred to as aIL10R) or an isotype control. Reports implicate autocrine IL10 in M2-like MΦ polarization^50,51^, and indicate that PI3Kγ inhibition, which elicited effects in vitro (Fig. 5), reduces IL10 production in TAMs^46^. Furthermore, we analyzed single-cell RNA sequencing data (scRNAseq) from patient biopsies in multiple cancer-types (OVCA, melanoma, head and neck squamous cell carcinoma, HNSCC), and found that IL10 signaling may occur at especially high levels in myeloid cells including TAMs expressing both IL10 and its receptor components IL10RA (Fig. 9a) and IL10RB (see “Methods”).

Although IL10 is reported to promote M2-like MΦ polarization, we confirmed aIL10R effects through two assays. We used the mannose receptor CD206/MRC1 as a marker of M2-like MΦ polarization, and confirmed that aIL10R reduced its expression in IL4-MΦ (Fig. 9b, Supplementary Fig. 17). We also quantified MΦ cellular morphology to assess MΦ polarization without relying on any single molecular marker (as similarly described^40^). Consistent with past studies^40^, round naive MΦ became elongated upon IL4 treatment, which partially reversed upon aIL10R treatment (Fig. 9c), thus suggesting aIL10R shifted MΦ polarization away from the M2-like phenotype.

**Fig. 10:**
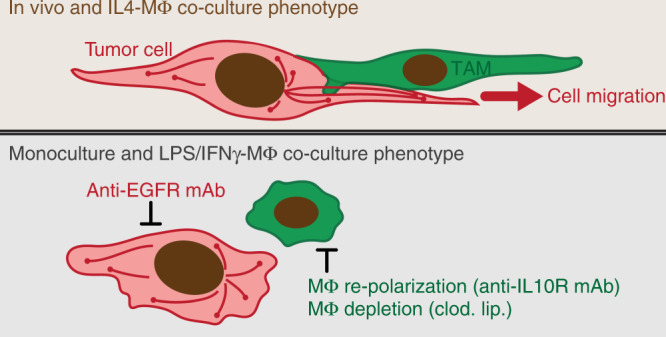
Summary schematic of TAM-dependent MT dynamics in cancer cells. (Top) Tumor cells in vivo exhibit high MT coherence and orientation along the major length axis of elongated, migrating cells. In vitro, cancer cells display this phenotype upon co-culture with M2-like IL4-MΦ. (Bottom) The IL4-MΦ co-culture phenotype depends on cell signaling pathways in both tumor cells and MΦ, including EGFR on tumor cells, and pathways known to influence MΦ polarization including PI3Kγ and IL10R. In vivo, treatment with an IL10R-neutralizing antibody reduces MT coherence and orientation in neighboring cancer cells.

In vitro, aIL10R blocked the effects of IL4-MΦ on MT coherence and orientation, as reflected by a shift in the PC describing the two features (Fig. 9d, calculated as in Fig. 5a; Supplementary Fig. 17), and also reversed the effects of IL4-MΦ on tumor cell circularity (Fig. 9e). Encouragingly, similar impacts of aIL10R on MT dynamics and cell shape were also observed in vivo by IVM (Fig. 9f–i, Supplementary Movies 1 and 2; measured as in Fig. 1). Overall, these data show how TAM polarization via IL10 locally shapes neighboring tumor cells to promote behaviors that distinguish in vivo MT dynamics from their in vitro counterparts (Fig. 10).

## Discussion

In this report, we examined a spectrum of MT dynamic features across thousands of individual MTs and applied multivariate statistical methods to interpret patterns in MT behavior across experimental conditions. Our studies in two cancer cell models revealed cellular MT coherence and alignment were central features of in vivo tumor cell motility dynamics, and surprisingly were modulated by pro-tumor MΦ signaling. The relevance of MT alignment extends to directional signaling, protein trafficking, and cellular migration. As examples, aligned MTs coupled with the endoplasmic reticulum facilitate fluid flow of cytoplasmic contents^52^, and MTs grow at the cell leading edge while depolymerizing at the trailing edge during cellular elongation and migration in angiogenesis and wound healing^53^.

How do TAMs shape tumor MT dynamics? Our data implicate MΦ polarization influenced by IL4, aIL10R, and PI3Kγ-targeted IPI-549, which in turn likely affects MT dynamics through interdependent processes, including (1) local signaling; (2) guidance through contact and spatial confinement; and (3) alignment through matrix remodeling and force generation. Altered transcription, cytoskeletal shape, and post-translational activities in polarized MΦ all affect these processes. Our in vitro data suggests contributions from the latter (MT dynamics decouple from gross cell shape changes within minutes in Fig. 8), and broadly establishes a role for TAM polarization. Experiments with erlotinib (Tarceva) and the human EGFR-neutralizing mAb225 (non-humanized C225, cetuximab/Erbitux) suggest ligand-dependent EGFR signaling on cancer cells is required. EGFR ligands including heparin-binding EGF (HB-EGF) are produced by both TAMs and cancer cells^41,54^. However, impacts of TAM polarization on ligand production are complex, and tumor EGFR signaling may likewise influence TAM polarization^55^. Thus, future work is needed to dissect EGFR-mediated tumor-TAM feedbacks, and more broadly, which other mechanisms are most important.

A majority of previous studies tracking MT end-binding proteins have relied on in vitro models and non-mammalian model organisms. However, in vivo mammalian MT dynamics have been examined in the nervous system using YFP-EB3 reporter mice, for instance, finding MT orientation and anterograde directionality along the neurite major axis^56^. Polarized MT dynamics are essential for neurite elongation and remodeling after injury or neurodegeneration^57^. Intriguingly, microglia and astrocytes bearing functional relationship to TAMs have been implicated in guidance of MT-rich axons^58^. Our results suggest that the polarization state of such cells—for instance as mediated by IL10R—could impact axon guidance, and IL10R signaling within astrocytes and microglia has been reported^59,60^.

Technical limitations of our approach must be appropriately weighed. Variable MT density within individual cells makes it difficult to uniformly capture all MT dynamics, and therefore, our analysis was focused on a subpopulation of more centrally located MTs. Comets transiting in and out of the focal plane may not form continuous tracks. This occurs in cells grown in 2D and especially in 3D environments, and similar challenges arise in cell shape analysis. Strict tracking supports low false positive rates, but may decrease abilities to detect smaller and slower comets resulting in higher false negative rates, and different imaging conditions may bias the subpopulations of MTs being tracked (Supplementary Fig. 2). We addressed many such issues through control experiments (for instance using synthetic imaging noise). Most importantly, this was addressed by testing hypotheses, including that TAM-repolarization via aIL10R may impact MT dynamics, through a comparison with matched control tumors or cells imaged under otherwise identical conditions. In this example, limitations and caveats of in vivo analysis applied equally across treatment groups, and MT dynamics still were observed to change in a manner consistent with the in vitro co-culture data.

Here we demonstrate how local microenvironmental signaling with TAMs can be as much of a distinguishing factor as the 3D extracellular matrix environment in affecting MT behavior in vivo compared to in vitro. In fact, MΦ polarization is likely playing a substantial role in shaping the extracellular matrix^61^. Our findings may be especially important for prodrug formulations of MT-targeted therapies based on NPs (e.g., Genexol PM, used clinically in Korea) and antibody-drug conjugation (e.g., trastuzumab emtansine/Kadcyla), as these can accumulate in TAMs^24,62^. Future studies examining MTs and MT-targeted drugs in vitro might evaluate their action in the context of relevant populations including TAMs to better gauge true in vivo effects.

## Methods

### Materials

HT1080, ES2, and Raw264.7 (Raw-MΦ) were originally from ATCC, and were cultured according to provider guidelines using DMEM (for HT1080 and Raw-MΦ) or McCoy’s 5a (ES2) medium, and 10% FBS (Atlanta Biologicals; HI FBS for Raw-MΦ), 100 IU mL^−1^ penicillin,100 μg mL^−1^ streptomycin (Invitrogen), with incubation at 37 °C and 5% CO_2_. Cells were routinely evaluated for mycoplasma contamination. HT1080-mem-mApple, HT1080-EB3-mApple, ES2-EB3-mApple, and ES2-mClover cells were all generated by lentiviral transduction. The construct mApple-EB3-7 was a gift from Michael Davidson (Addgene plasmid # 54892), and mClover expression construct (pLentiCMV Puro DEST ERKKTRClover) was a gift from Markus Covert (Addgene plasmid # 59150; http://n2t.net/addgene:59150]; RRID:Addgene_59150)^63^. Cell lines were tested for mycoplasma contamination using Lonza MycoAlert. Negative test results were obtained for cell lines used in the study. Fluorescent-conjugated nanomaterials were synthesized^24,48^ using PacificBlue (λ_ex_/λ_em_ = 401 nm/452 nm) or VT680XL (λ_ex_/λ_em_ = 665 nm/688 nm) as labeling fluorophores using previously described protocols^24,48^. Briefly, carboxymethylated polyglucose (550 mg, 2.3 mmol COOH) was dissolved in 6.2 mL of MES buffer (50 mM), followed by addition of EDC (2.4 g, 12.5 mmol) and NHS (457.2 mg, 4.0 mmol). After 10 min at room temperature (RT), L-lysine (401.8 mg, 3.5 mmol) dissolved in 0.7 mL MES buffer (50 mM) was mixed with the solution for 5 h at RT. The mixture was then slowly added to ethanol (30 mL). After centrifugation (2500 × *g*) for three minutes, a white pellet formed. This pellet was then dissolved in Milli-Q H20, filtered using a 0.22 μm nylon syringe filter (Thermo Fisher), filtered again with a centrifugal filer (MWCO 8–10 kDa, Amicon Ultra), and finally lyophilized resulting in solid particles (Macrin-NP). The Macrin-NP (20 mg) was then conjugated to VT680XL by dissolving it in MES buffer (200 μL, 50 mM) followed by triethylamine (2 μL, 14.4 μmol) and VT680XL-NHS (0.5 mg in 215.5 μL DMF, 0.27 μmol). The solution was shaken at RT for 3 h, purified using PD-10 columns with water, and filtered using centrifugal filters. This new solution was further diluted with MES buffer (200 μL, 50 mM, pH 6.0) and treated with Et3N (2 μL) and succinic anhydride (100 μL, stored in 750 mM in DMSO). After shaking this mixture overnight, VT680XL-Macrin was purified using PD-10 columns and centrifugal filters (MWCO 10 kDa, Amicon Ultra). Fluorescently labeled Macrin was analyzed and confirmed by an SEC, a Varian Cary 100 UV/vis spectrophotometer, and a Varian Cary Eclipse fluorescence spectrometer. Poly(D,L-lactic-co-glycolic acid) PLGA-BODIPY encapsulated Poly(D,L-lactic-co-glycolic acid)-b-poly(ethylene glycol) PLGA-PEG polymeric micelles and angioSPARK-680 were used as long-circulating vascular labeling agents in Figs. 3 and 7, respectively^24^.

The following antibodies and drugs (listed as target; source; calatog/clone#; concentration) were used for in vitro experiments only: Y-27632 (ROCK inhibitor; Tocris; Cat # 1254; 10 μM); serabelisib (PI3Kα inhibitor; Selleck Chemicals; Cat # S8581; 0.5 μM), AZD6482 (PI3kβ inhibitor; Select Chemicals; Cat # S1462; 0.5 μM), AMG319 (PI3Kδ inhibitor; Selleck Chemicals; Cat # S7813; 0.5 μM), IPI-549 (PI3Kγ inhibitor; Selleck Chemicals; Cat # S8330; 0.5 μM), erlotinib (EGFR inhibitor; Selleck Chemicals; Cat # S7786; 5 μM), UNC2025 (MERTK inhibitor; Selleck Chemicals; Cat # S7576; 100 nM), R848 (TLR7/8 agonist; Selleck Chemicals; Cat # S8133; 1 μM), human Integrin β1/CD29 Antibody (R&D Systems; Clone # P5D2; 10 μg mL^−1^), anti-CSF1R (BioXCell; Clone # AFS98 ;10 μg mL^−1^), and Axl Fc Chimera (R&D Systems; Cat# 154-AL-100; 10 μg mL^−1^). For measuring MT dynamics, each treatment was given 2–4 h prior to imaging. For measuring cell shape each treatment was given 24 h prior to imaging. The following antibodies were used for in vitro and in vivo experiments: InVivoPlus anti-mouse IL-10R (anti-IL-10R; BioXcell; Clone # 1B1.3A; 10 μg mL^−1^), InVivoMAb anti-human EGFR (anti-EGFR; BioXcell; Clone # 225; 10 μg mL^−1^), InVivoPlus rat IgG1 isotype control (IgG ctrl; BioXcell; Clone HRPN; 10 μg mL^−1^), InVivoMAb mouse IgG1 isotype control (IgG ctrl; BioXcell; Clone MOPC-21; 10 μg mL^−1^).

### Intravital microscopy

Confocal microscopy was performed using an Olympus FV1000 multiphoton imaging system. Animals were used in accordance with guidelines from the Institutional Subcommittee on Research Animal Care and under approval from the Institutional Animal Care and Use Committee (IACUC) at Massachusetts General Hospital. Animals were housed in a light–dark cycle, climate (temperature and humidity via heating venting air conditioning, HVAC) controlled vivarium and kept under ad libitum food and water diet supplied by the MGH Center of Comparative Medicine staff. Two million parental HT1080, HT1080-EB3-mApple, HT1080-memApple, and/or ES2-EB3-mApple cells were suspended in 50 μL PBS, injected under the fascia 30 min after surgical dorsal window chamber implantation, in female 4–8 week-old nu/nu mice (Cox7, MGH), and imaged ~2 weeks later with visible tumor formation. Static and time series images were collected using a XLUMPLFLN 60× or 20× water immersion objective (NA 1.0; Olympus America) and up to 10× digital zoom. Images were scanned sequentially using 405-, 473-, 559- and 633-nm diode lasers in combination with a DM405/488/559/635-nm dichroic beam splitter. Emitted light was then separated and collected using appropriate combinations of beam splitters (SDM473, SDM560, and SDM 640) and emission filters BA430-455, BA490-540, BA575-620, and BA655-755 (all Olympus America). For EB3 imaging, to minimize photobleaching and potential artifact from translational drifting, we captured brief snapshots of MT dynamics for a given cell: movies were acquired for 40–130 s in duration with a frame rate of between 0.8 and 2.7 s per frame, a relatively short dwell time (8 µs pixel^−1^), low laser power (λ_ex_ 559 nm, 2% power), and at a single plane of focus. In instances (Fig. 9), aIL10R or rat IgG isotope control was administered i.p. (200 μg in 100 μL of PBS) to mice 5–7 days after HT1080-EB3-mApple injection and treated again after 2 days. Images were acquired after the first and second treatments. For visualizing TAMs, 24 h prior to the first day of imaging, mice were administered VT680-labeled polyglucose NPs (Macrin)^48^.

### Study design

Sample sizes were determined by counting the number of MT tracks, cells, tumors, or mice depending on the analysis. For track-based analyses sample sizes were not predetermined as all the tracks computationally determined were used for downstream analyses. Sample sizes for cells, tumors, or mice were chosen based on estimated effect sizes from prior studies with this xenograft model and in vitro cell culture experiments^19,20,23,24,64^. All experiments were performed with ≥2 independent replicates as described in the figure captions. Assignment of animals into treatment groups, as with the in vivo IL10R experiments, was performed randomly. For both in vivo and in vitro experiments, samples that underwent different treatments (drug treatment, co-culture conditions, MΦ polarization, etc.) were placed into different groups. For all MT track and shape experiments, results were obtained via unbiased computational scripts, and image acquisition was performed with unbiased parameters constant across treatment groups.

### Disseminated OVCA imaging

Five million ES2 cells stably transduced to express the GFP-variant, mClover, were suspended in 200 μL PBS and i.p. injected into female nu/nu mice of 6–10 weeks age to establish a model of disseminated OVCA. Beginning 3 days after tumor inoculation, mice were treated i.p. with 150 μL clod-lip (5 mg mL^−1^) or PBS control liposomes (Liposoma BV). Three and 6 days after, 50 μL clod-lip or PBS-lip were again used. The following day, PacBlue-labeled polyglucose NPs (Macrin) were administered i.v. and 24 h later, mice were sacrificed for immediate ex vivo confocal imaging of tumor-bearing organs. Macrin has been shown by flow cytometry and imaging to be >90% selective for TAMs in mice^48^.

### BMDM culture

Murine bone marrow-derived MΦ (BMDMs) were isolated from the femurs and tibias of 6–8-week-old C57BL/6 mice. Marrow was flushed from the bones using 10 mL cold PBS flush via 21-gauge needle, cells were centrifuged for 5 min at 300 × *g*, and resuspended with PBS. Ammonium chloride (0.8%, NH_4_Cl, StemCell Technologies) was added at 4 °C for 5 min to lyse red blood cells. The mixture was centrifuged again for 4 min at 300 × *g*. Cells were then plated on a 24-well plate with Iscove’s Modified Dulbecco’s Medium (IMDM), 10% PBS + P/S, and 10 ng mL^−1^ MCSF (Peprotech). The medium was replaced every 2 days for 6 days. On day 7, MΦ were cultured in fresh IMDM (MCSF-MΦ) or further polarized by replacing MCSF with 100 ng mL^−1^ lipopolysaccharide and 50 ng mL^−1^ interferon-gamma (LPS/IFNγ-MΦ), or 10 ng mL^−1^ interleukin-4 (IL4-MΦ; Peprotech). After 24 h media was replaced with fresh IMDM, 10% PBS + P/S for 12 h, MΦ treated 8 h with 10 nmol Alexa647-labeled Macrin, and then treated with fresh IMDM.

### BMDM and tumor cell co-culture

Immediately following the steps outlined above, ~6500 HT1080-mApple cells or 10,000 ES2-EB3-mApple cells were seeded into wells already containing polarized MΦ, where they were then co-cultured for ~48 h in a 96-well plate (Ibidi). The co-cultures and matched monocultures were all grown in IMDM, and then fixed using a 4% PFA + PBS for 20 min and imaged. For conditioned media experiments, ~6500 tumor cells were seeded per well into a separate 96-well plate. The media from the MΦ culture was transferred into the separate well plate containing newly seeded tumor cells. Tumor cells were grown for 48 h, followed by fixing and imaging as above. In vitro images for cell shape quantification and ES2-EB3 imaging were acquired using a modified Olympus BX63 inverted microscopy system with environmental chamber and robotic stage.

### RAW264.7 and tumor cell culture

Approximately 500 RAW264.7 were seeded in a 96-well plate and treated with/without 20 ng mL^−1^ IL-4 (24 h), followed by VT680 labeled Macrin (24 h). On day 3, ~1000 HT1080-EB3-mApple cells or ES2-EB3-mApple cells were seeded onto the plate. Cells were co-cultured for 24 h followed by drug treatment lasting 2–4 h or 24 h (except aIL10R, which was treated for 48 h prior to imaging; Fig. 9e). For ES2-EB3 MT dynamics drug screen, RAW264.7 were polarized and labeled with Macrin prior to seeded on a 10 mL petri dish, and were seeded simultaneously with tumor cells.

### 3D monoculture

Three-dimensional cultures of cancer cells were performed according to previously described protocol^65^. Briefly, HT1080 EB3-mApple cells were suspended in a 2.5 mg mL^−1^ collagen I gel extracted from rat tail (Corning) at the density of 1 million cells mL^−1^. The collagen gel containing the cells was deposited onto a MatTek dish (MatTek Corp.), and allowed to polymerize at 37 °C and pH 8 for 30 min. in a humidified chamber. Following the polymerization, fresh growth media was introduced into the MatTek dish. The cells were imaged with confocal microscopy for MT dynamics following overnight incubation (24 h) in a humidified incubator.

### Western blot for studying MΦ polarization

RAW264.7 were cultured in six-well plates and treated with ±20 ng mL^−1^ IL-4, ±10 μg mL^−1^ aIL10R, and/or ±10 μg mL^−1^ Rat IgG isotope control, resulting in a total four conditions (+IL-4 +aIL10R, +IL-4 +IgG, +aIL10R, +IgG). Cell lysates were extracted from each well with RIPA buffer containing protease inhibitor and PMSF. Thirty micrograms of total protein was resolved on 4–12% NuPAGE electrophoresis gels (Invitrogen) and then transferred onto nitrocellulose membranes (Invitrogen). The membranes were incubated with the following antibodies at the corresponding dilution factors: rabbit anti-CD206 antibody (Abcam, polyclonal) at 1:1000 or mouse anti-β actin antibody (Cell Signaling Technology, clone#8H10D10) at 1:2000. This was followed by incubation with the appropriate secondary antibodies conjugated to horseradish peroxidase (Cell Signaling Technology). The immunoreactive bands were detected with ECL Chemiluminescent substrates (Thermo). To quantify western blot images, densitometry analysis was performed using ImageJ, and the densitometry value for each protein was normalized to β-actin before further being normalized.

### MT tracking and feature extraction

EB3 comets were detected and linked to form MT tracks using the U-track software ([http://www.utsouthwestern.edu/labs/danuser/software/])^25^. Cell boundaries, in the form of a mask, were constructed using ImageJ. In order for a MT track to be successfully called, it must pass a strict set of filters: (1) be present in a marked cell, (2) comets must be detected in at least three frames, (3) only a maximum of one gap is present, (3) must travel at least 0.5 µm, and (4) comet persistence must be >0.60. Persistence was measured on a scale of 0–1, where 1 indicated a straight track. These constraints were enforced either directly using the U-track software or a custom script implemented in Python (Python3.6) that required a track CSV file, cell mask, and image metadata containing the image frame rate and resolution as input. The following U-track parameters and the defined ranges were used to achieve accurate tracking: Low-pass gaussian st. dev (1–3 pixels); high-pass gaussian st. dev (4–5 pixels); watershed segmentation minimum threshold (2–3 st. dev); minimum track length after first frame (2–4); minimum number of gaps (0–1). The remaining parameters were set to the default settings. After obtaining a resolved set of MT tracks, 14 features for each track were calculated:

(1) Average speed (µm s^−1^), whereby the distance traversed between each frame was calculated via the python numpy library gradient function, and averaged across the entire time-lapse for a given track; (2) minimum speed, (3) maximum speed, and (4) speed standard deviation for a given track across frames; (5) net displacement (µm), calculated as |track end coordinate—track start coordinate|; (6) path length (µm), calculated as the total distance traversed by a track; (7) persistence, defined by (net displacement) × (path length)^−1^; (8) curvature, calculated as a third degree polynomial fit to the MT track using the numpy python library polyfit function. From this fitted polynomial, the curvature between frames was computed using Eq. 1.

1 $$\kappa = \frac{{\left| {\frac{{dx}}{{dt}}\left( {\frac{{dy}}{{dt}}} \right)^2 - \frac{{dy}}{{dt}}\left( {\frac{{dx}}{{dt}}} \right)^2} \right|}}{{\left( {\left( {\frac{{dx}}{{dt}}} \right)^2 + \left( {\frac{{dy}}{{dt}}} \right)^2} \right)^{\frac{3}{2}}}}.$$

*x* and *y* are the coordinates of the MT comet at each frame. Curvature was computed at each *x*–*y* coordinate and all values were averaged, resulting in one curvature value per track; (9) distance to cell edge, defined by a distance transform image (DT) created from the inverse of the cell mask. The pixel value in the DT image corresponds to the distance from the cell edge. This measurement was normalized to be independent of absolute cell size by dividing the sum of all pixel values that fall within the cell boundaries from the DT image by the total number of pixels inside the cell. Min–max normalization was lastly performed to scale measurements between 0 and 1; (10) distance to the cell major axis was calculated using the regionprops function from the sklearn python package, via the orientation, major_axis_length, and centroid parameters. From these parameters, points on the cell major axis were identified. Distances were normalized by the farthest point from the major axis along the cell boundary, such that all track distance measurements fell between 0 and 1; (11) distance to the cell minor axis was computed and normalized similar to the above, with the minor axis defined as perpendicular to the major axis; (12) local track coherence, calculated by the cosine of the angle between a query track and tracks within 20 μm radius within the same cell, and averaged across all such pairs of tracks; (13) cellular track coherence, calculated similarly to the local track coherence but for all tracks of a given cell rather than within a given radius; (14) track orientation, calculated as the cosine of the angle between the query track and the cell major axis. All features were calculated using python scripts. Track visualizations were constructed using either python scripts or GraphPad Prism (Prism 8) software.

To test statistical robustness against unevenly distributed MT track behaviors from cell to cell, a permutation test was performed such that cell labels were shuffled between the different groups. A naive wilcoxon rank sum test between pairs of cell populations was applied to determine the permutation *p* value, for each MT feature. To determine the final *p* value for each feature, the number of permutations with a *p* value less than the *p* value of the true distribution was divided by the total number of permutations. Without any permutation, the reported *p* value obtained from a naive wilcoxon rank sum test was <10^−40^ in some cases. A maximum of 1000 permutations were run for each experiment. If there were no permutations that had a lower *p* value than the naive rank sum test, then the final *p* value is reported as *p* = 0.001.

Effect size calculations between two different MT populations (i.e., in vivo, in vitro 2D, etc.) was determined using Cohen’s D statistic, measured by Eq. 2. SD_pooled_ is measured by Eq. 3.

2 $${\mathrm{Cohen}}\prime {\mathrm{s}}\,{\mathrm{D}} = \frac{{\mu _1 - \mu _2}}{{\rm{SD}_{pooled}}}.$$

3 $${{\rm{SD}}}_{{\rm{pooled}}} = \frac{{\sqrt {{{\rm{Var}}_1 + {\rm{Var}}_2}} }}{2}.$$

The means of population 1 and 2 are represented by μ_1_ and μ_2_, and the variances are represented by Var_1_ and Var_2_, respectively.

Additional tests were conducted to determine the robustness of the analysis. The first test ensured that incompletely imaged cells in vivo did not affect the significance tests. Cells that were more than 20% outside the field of view were omitted during this test and a two-tailed permutation comparing in vivo and in vitro HT1080-EB3-mApple cell populations was conducted as described above (Supplementary Fig. 6). The second test ensured that significance between in vivo and in vitro populations was not the result of noise differences or image quality. Artificial noise was added to a batch of in vitro cells (Gaussian noise with 0 mean and 100, 200, and 400 SD in pixels) using the ImageJ add specified noise function. The effect size between the noisy cell populations and the in vivo cell populations was measured. Significance was also measured between the artificially noisy and in vitro cells with no artificial noise added (Supplementary Fig. 7).

### PC analysis of MTs

PCA of MT dynamics from the in vivo, in vitro 3D, and in vivo cell populations was performed using the python scikit-learn package on all 14 of the MT features. Outlier tracks, or tracks where any of the feature values were not within 5 and 95 percentile were removed prior the PCA. Furthermore, the naive distribution of several features were not normal and highly skewed. Therefore, several transformations were used depending on the feature type. For features with a right skewed distribution, a log transformation (curvature, displacement, path length, average speed, minimum speed, maximum speed, and speed SD) or sqrt transformation (major axis distance and minor axis distance) was used. For features with a left skewed distribution, sqrt(1–x) transformation (MT orientation and persistence) was used. For the remaining features, no transformations were performed. The transformed dataset consisting of approximately gaussian distributed features were normalized (mean = 0, SD = 1).

### PCA analysis of select MT features, Clustergram, and K–L Divergence

PCA was performed using the python scikit-learn package. Two features, track orientation and track cellular coherence, were used, such that PC1 is a linear combination of the track orientation and cellular coherence features. To examine the differences between two track features: cellular coherence and orientation, across all nine HT1080 cell populations or conditions, K–L divergence was computed between the PC1 distributions of each condition (Supplementary Fig. 11). Clustergram analysis involved finding the average MT track feature value for each HT1080 tumor cell population. This was accomplished by first removing all MT tracks that had feature values greater than three SD’s away for any of the plotted features of interest, followed by averaging track feature values for each cell population/condition. All columns were normalized (0 mean, 1 SD). The average values were then clustered using hierarchal clustering (method=complete, metric=euclidean) via the clustergram function available in the python seaborn package.

### Cell shape and contact analysis

Cell contact and shape analyses were implemented using MATLAB (MATLAB_R2016B) and/or Python. Masks of tumor cells and/or MΦ were obtained via manual curation or using a custom Cellprofiler pipeline. From the tumor cell masks, the computed cell circularity was determined using the following equation: 4*πAP*^−2^, where *A* is the area of tumor cell and *P* is the perimeter of the tumor cell. To standardize cell circularity measurements, cell circularities for cells grown under the control conditions were normalized (0 mean, 1 SD), while cell circularities for cells grown in other conditions were transformed according the pre-normalized circularity measurements from the control condition. In addition to circularity, cell eccentricity was also calculated from the MATLAB or python skimage regionprops function. From the MΦ mask, a distance transform image (where each pixel value is the distance to the nearest MΦ) was constructed. The minimum distance between tumor cell and MΦ was computed using the distance transform and the tumor cell mask. Tumor cells were binned according to low, medium, or high unnormalized circularity measures (Fig. 6e: low = 0,0.4), medium = [0.4,0.7), high = [0.7,1), Fig. 6g: low = [0,0.7), high = [0.7,0.8), medium = [0.8,1)). Computing significance scores over individual cells resulting in extremely low significance values, and therefore, significance was calculated using cell averages from image replicates for in vitro cells and tumors for vivo cells in all cell shape analyses unless explicitly stated otherwise. To calculate *p* values, averages across batches were calculated and outlier less Q1 − 1.5*IQR(inter quartile range) or greater than Q3 + 1.5*IQR were excluded from the calculations. A two-sided *t* test or nonparametric wilcoxon rank sum test was then applied to obtain a final *p* value.

### Cell shape PCA analysis

For cell shape PCA analysis, cells were automatically segmented via a custom CellProfiler script, generally using DAPI and/or wheat germ agglutinin (WGA; Thermo Fisher; Cat # W11261) staining or segmentation using HT1080-memApple (Fig. 6). Once cell masks have been determined (stored a labeled image), a custom MATLAB script was used to extract the following shape parameters: circularity, extent, solidity, convexity, eccentricity, area, and perimeter. Calculations for each of these shape features was performed using the MATLAB regionprops function. Following construction of a cell by feature matrix, a custom python script was used to scale the data (mean = 0; SD = 1) and run PCA, as well as calculate the loadings and population averages (Figs. 2d, 6a, and 8e).

### Cell migration analysis

HT1080-mem-mApple cells were imaged by IVM as described above with the EB3-mApple model and in a prior report^24^. Briefly, to track cell migration, confocal z-stacks were acquired roughly every 5–10 min at multiple tumor locations for ~2 h as reported^24^. Tumor-associated phagocytes and microvasculature were labeled by co-administration of a dextran-coated NP known to be highly phagocytosed by perivascular macrophages (ferumoxytol-VT680XL), along with a more slowly extravasating PLGA-PEG polymeric NP^24^. Ferumoxytol-VT680XL was intravenously co-administered (750 μg Fe) with PLGA-PEG NP (100 nM BODIPY) immediately prior to time-lapse imaging. Fifty cells were identified, and a cell mask was created for each cell. From the cell mask, morphological features of eccentricity and circularity were calculated using custom python scripts. To calculate migration rates for each of these 50 tumor cells, the center of the cell was approximated and traced over a minimum of four frames.

### TAM and vasculature association analysis

The association between HT1080-mem-mApple cells and vasculature in vivo (Fig. 3C) was determined by first generating a mask image representing the tumor vasculature via Otsu thresholding on the original images labeled with either AngioSPARK-680 or PLGA-PEG polymeric NP. Individual cell masks were manually identified. If any region of the tumor cell overlapped with the vasculature mask, then the TC is marked as contacting vasculature. The same process was applied to macrophage images to study macrophage-tumor cell association.

### Monoculture and IL4-MΦ co-culture effect size/significance analysis

The effect sizes between HT1080-EB3-mApple and ES2-EB3-mApple cells grown in monoculture and in co-culture with IL4-polarized MΦ was determined for all 14 MT features. The effect sizes were computed separately for each experiment run (2 for ES2-EB3-mApple) and (4 for HT1080-EB3-mApple) to account for potential batch effects, and pooled together. Some runs had slightly varying conditions described as follows: ES2-EB3-mApple runs 1 and 2: untreated monoculture vs IL4-polarized BMDM MΦ co-culture. For HT1080-EB3-mApple cells: (1) untreated monoculture vs IL4-polarized BMDM MΦ co-culture, (runs 2–4) monoculture and IL4-polarized Raw MΦ; both treated with either Rat IgG isotope control (run 2), mouse IgG isotope control (run 3), or DMSO control (run 4). Effect sizes for each run were calculated using Cohen’s D Statistic (see “Methods”: MT tracking and feature extraction) and averaged across multiple runs. To generate violin plots (Fig. 4D), ES2-EB3-mApple tracks were combined from both runs (top) and HT1080-EB3-mApple tracks were combined for runs 2–4 (bottom). To calculate the significance of each feature between monoculture and co-culture conditions, tracks from each batch were individually normalized (mean = 0, st. dev.  = 1). An equal number of cells was randomly sampled (33 HT1080 cells and 33 ES2 cells), and a maximum of 25 tracks from each cell was subsampled. Thousand permutations were then performed for two pooled MT track populations (±MΦ co-culture) such that cell labels for MT tracks were shuffled between the cell populations being compared. A naive wilcoxon rank sum test between pairs of cell populations was applied to determine the permutation *p* value, for each MT feature. For each feature, the number of permutations with a *p* value less than the *p* value of the true distribution was divided by the total number of permutations to obtain a permutation based *p* value. For multiple hypothesis testing correction (12 different features), a Benjamini–Hochberg correction (Q(fdr) = 0.05) from the python statsmodels package was used to generate the final *p* value.

### Single-cell RNA-sequencing (scRNAseq) analysis

Analysis of ligand-receptor interaction from scRNAseq data was performed based on prior methods^66^. Briefly, scRNAseq data from biopsied patients was pooled across individuals. Cell-type identities (tumor, T-cell, myeloid cell, etc.) were provided from the published data annotations, and the average expression of IL10 was multiplied by the average expression of IL10RA or IL10RB for all such cell-type populations. Values were normalized to the maximum observed product (scaled to a maximum of 1.0). SPRING software (accessed Dec 2019 - Jan 2020) was used for dimensionality reduction to visualize and categorize key cell populations^67^. For OVCA, scRNAseq data (GSE118828) was pooled across samples from nine biopsied subjects, including five with high-grade serous OVCA, 2 with low-grade serous OVCA, 1 with metastatic peritoneal disease, and 1 with a benign lesion^68^. Other analyzed data included scRNAseq data from 19 melanoma patients (GSE72056)^69^ and 18 patients with head and neck squamous cell carcinoma (HNSCC; GSE103322)^70^. In all three datasets, IL10 to IL10RB signaling was highest for homotypic MΦ (MΦ to MΦ signaling) compared to all other cell-type pairings.

### Reporting summary

Further information on research design is available in the Nature Research Reporting Summary linked to this article.

## Supplementary information

Supplementary Information

Description of Additional Supplementary Files

Supplementary Movie 1

Supplementary Movie 2

Reporting Summary

## Data Availability

The source data underlying all main figures (Figs. 1d–f, 2b–e, 3c, d, 4a–d, 6a–c, e, g, h, 7a, c, f, 8a–f, 9a–f, h, i) and supplementary figures are provided as a Source Data file. All additional data that support the findings of this study are available from the authors on reasonable request. GSE accession numbers for publicly available data used in this study are GSE118828, GSE72056, GSE103322. Kaplan–Meier survival curves were derived from cBioportal (https://www.cbioportal.org/) which uses data from the Cancer Genome Atlas (TCGA). Source data are provided with this paper.

## Source data

Source Data

## Code availability

Scripts along with example images and data formats for extracting MT features are available online at https://github.com/gluthria/MT_Dynamics. All additional code used in this study are available from the authors on reasonable request. Source data are provided with this paper.
