# Supplementary material for: In vivo microscopy reveals macrophage polarization locally promotes coherent microtubule dynamics in migrating cancer cells
Source: Nat Commun. 2020 Jul 14;11:3521. doi: 10.1038/s41467-020-17147-y (PMC7360550; doi:10.1038/s41467-020-17147-y)
Supplement: Supplementary file 1 — Supplementary Information [file 41467_2020_17147_MOESM1_ESM.pdf]

Supplementary Information, Luthria et al.

In vivo microscopy reveals macrophage polarization locally promotes coherent microtubule dynamics in migrating cancer cells.

Gaurav Luthria[1,2], Ran Li[1], Stephanie Wang[3], Mark Prytyskach[1], Rainer H. Kohler[1], Douglas A. Lauffenburger[3], Timothy J. Mitchison[4], Ralph Weissleder[1,4,5], Miles A. Miller[1,5]

1. Center for Systems Biology, Massachusetts General Hospital Research Institute, Boston, MA, 021
2. Department of Biomedical Informatics, Harvard Medical School, Boston, MA 02115
3. Department of Biological Engineering, Massachusetts Institute of Technology, Cambridge, MA 02181
4. Department of Systems Biology, Harvard Medical School, Boston, MA 02115
5. Department of Radiology, Massachusetts General Hospital and Harvard Medical School, Boston, MA 02115

*email:
rweissleder@mgh.harvard.edu
miles.miller@mgh.harvard.edu

This PDF File includes:

Supplementary Figure 1. Visualizing *in vivo* and *in vitro* MT tracks.
Supplementary Figure 2. Correlations between EB3 and cancer cell behavior.
Supplementary Figure 3. Visualization of individual MT features.
Supplementary Figure 4. Quantifying MT dynamics in ES2 xenografts.
Supplementary Figure 5. Measuring MT dynamics independent of cell shape.
Supplementary Figure 6. Permutation testing of MT track statistics.
Supplementary Figure 7. The effect of artificial image noise on MT track statistics.
Supplementary Figure 8. Parameter sensitivity analysis in MT tracking.
Supplementary Figure 9. Representative imaging of tumor cells in 3D culture.
Supplementary Figure 10. Individual track and cell-average distributions of MT features.
Supplementary Figure 11. Quantitative comparison of MT behavior under distinct culture conditions.
Supplementary Figure 12. Permutation statistics and individual track-level data in the ES2 co-culture model.
Supplementary Figure 13. ES2 MT dynamics in response to targeted reagents and drugs.
Supplementary Figure 14. HT1080 MT dynamics in response to targeted reagents and drugs.
Supplementary Figure 15. Correlations between cell shape and MT behaviors.
Supplementary Figure 16. Quantifying tumor cell shape in response to clodronate liposome treatment.
Supplementary Figure 17. The effect of Anti-IL10R antibody treatment on MT dynamics.
Supplementary Figure 18. Quantifying macrophage proximity and subcellular orientation to tumor cells.

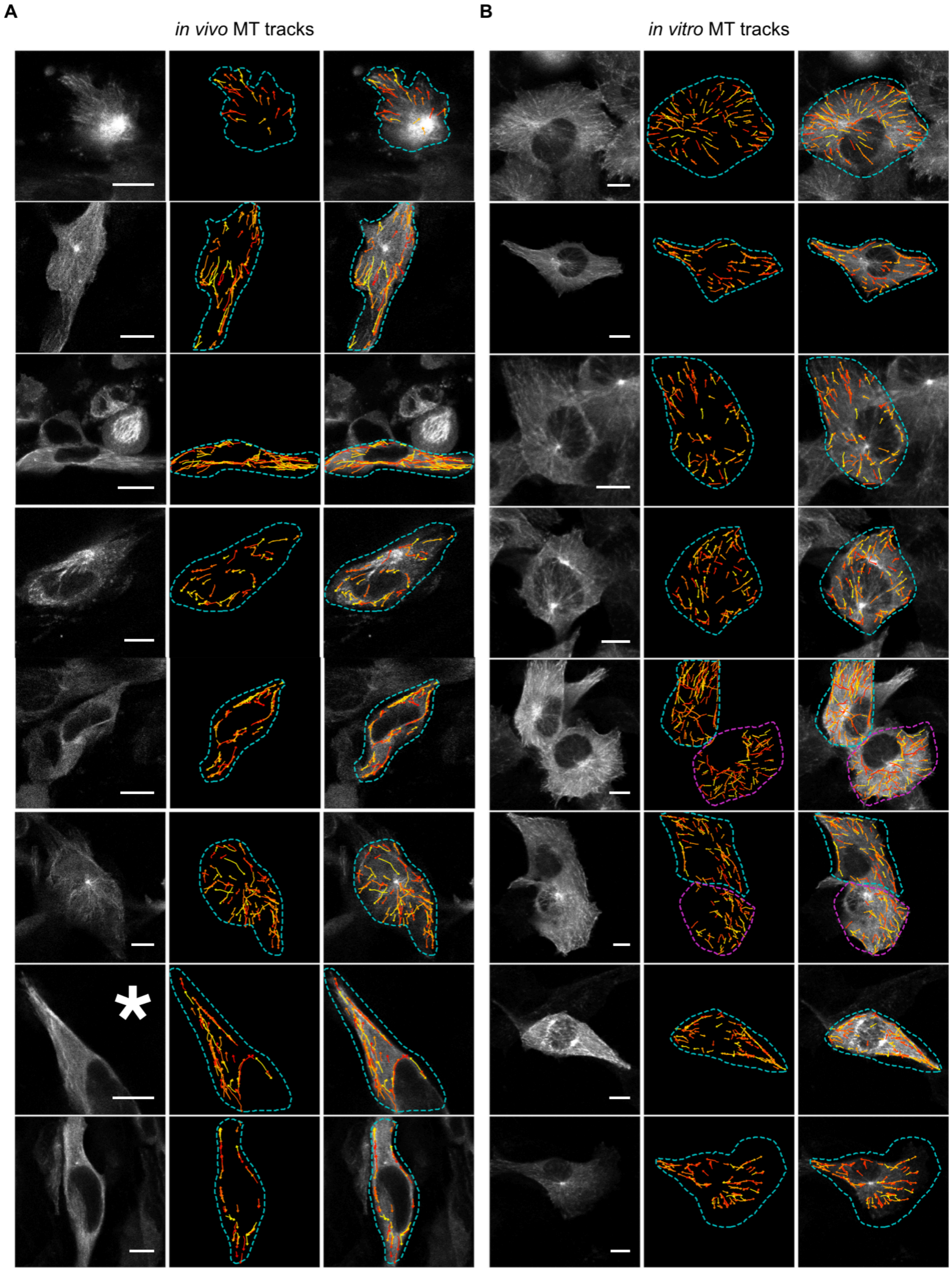

Supplementary Figure 1. Visualizing *in vivo* and *in vitro* MT tracks.
(A) Representative *in vivo* time-lapse images of HT1080 EB3-mApple cells growing in a dorsal window chamber were obtained via IVM (left). MT tracks were computationally identified (center) and randomly pseudo-colored from red to yellow. (B) Representative *in vitro* time-lapse images were obtained from HT1080 EB3-mApple cells in 2D culture using the same imaging system (left) and computational tracking software (center). (A-B) Scale bar, 10 μm. Asterisks mark cells excluded in Fig. 1 analysis due to incomplete cell imaging within the field of view. A total of n=34 *in vivo* cells and n=39 *in vitro* cells were analyzed.

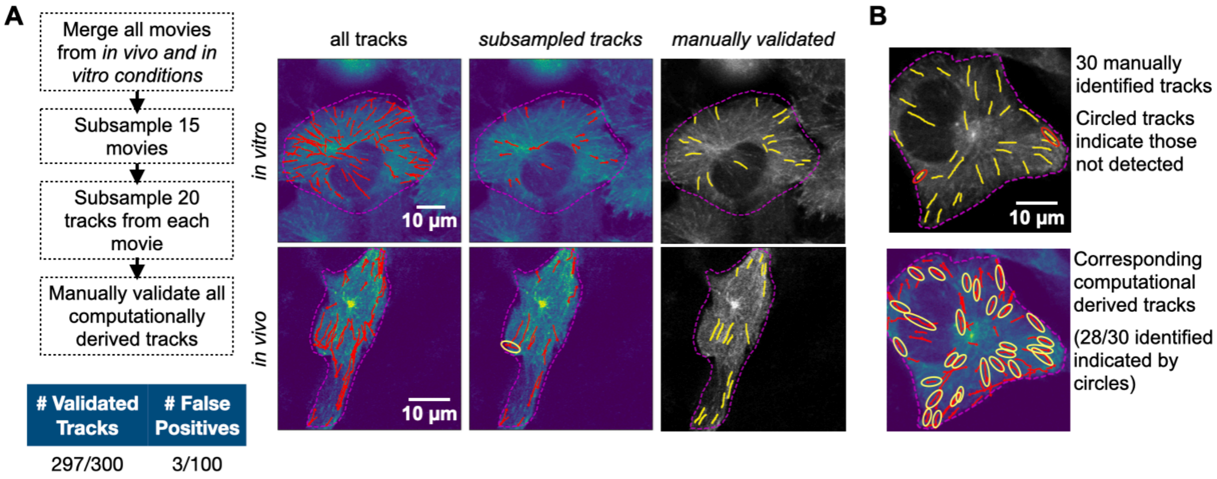

C

MT feature	R^2	P-value
Curvature	0.02	0.38
Edge Distance	0.05	0.15
Distance to Major Axis	0.01	0.46
Orientation to Major Axis	0.01	0.66
Distance to Minor Axis	0.05	0.17
Displacement	0.22	0.00
Pathlength	0.19	0.01
Persistence	< 0.01	0.90
Speed	< 0.01	0.86
Max Speed	0.00	0.79
Min Speed	< 0.01	0.91
Speed SD	< 0.01	0.97
Local Coherence	0.01	0.66
Cellular Coherence	0.01	0.55

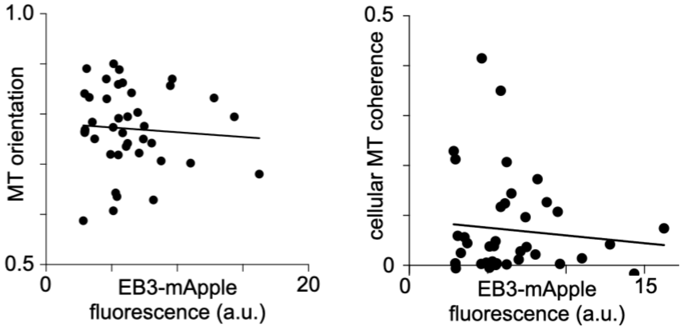

D

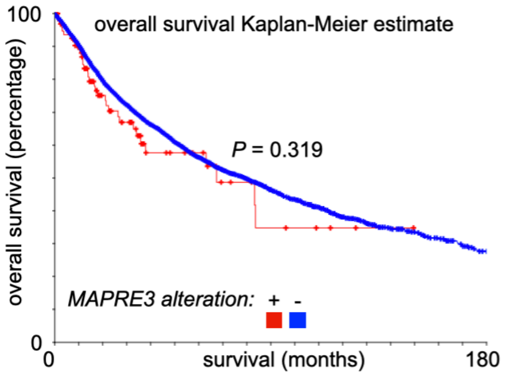

MAPRE3 alteration status	Total Cases	Deceased Cases	Median Survival
+	99	34	67 mo.
-	10703	3480	79 mo.

Supplementary Figure 2. Correlations between EB3 and cancer cell behavior. (A) Workflow (left), example case (middle), and statistics (right) for hand-validation of MT tracking to assess false positive rate. (B) False negative estimation is challenging as confirming the identity of all tracks is a difficult task. Nevertheless, we identified 30 of the most visible tracks in one representative *in vitro* movie and determined how many of these tracks were computationally detected and analyzed. **(C)** In HT1080 cells, EB3-mApple expression was examined for linear correlation with various MT features (Pearson's correlation coefficient; *two-tailed t-test; n=38 cells), with significant correlates highlighted in red. At right, corresponding cell-by-cell values for MT orientation and cellular MT coherence are shown (correlation not significant). **(D)** Kaplan-Meier analysis of overall survival across The Cancer Genome Atlas (TCGA) as a function of EB3 alteration (copy number amplification and mutation; P-value was calculating using using a two-sided log-rank test; cBioPortal.org).

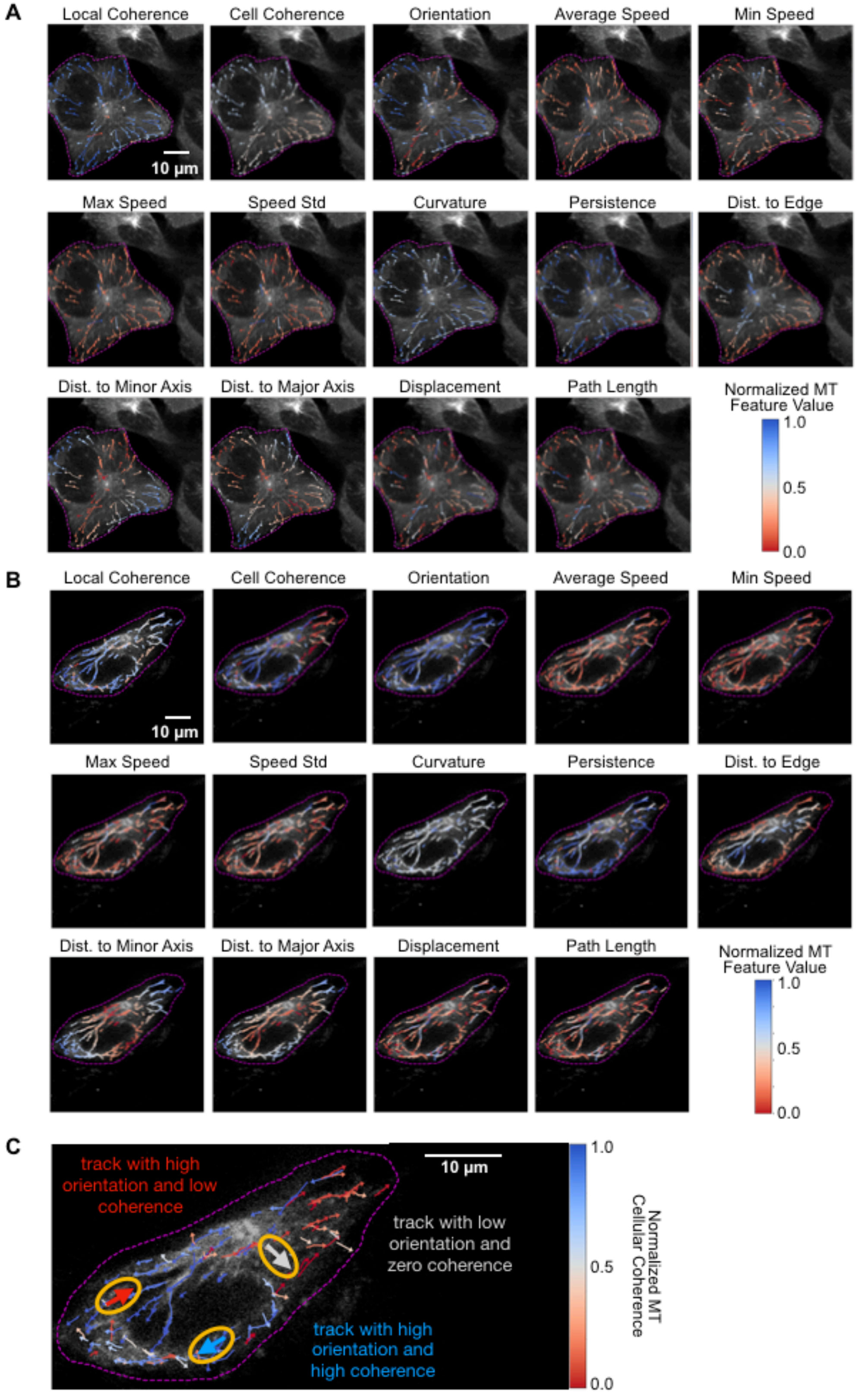

Supplementary Figure 3. Visualization of individual MT features.
(A) A representative HT1080 EB3-mApple cell grown in 2D culture is shown to visualize 14 MT track features. MT tracks are color coded based on the indicated track feature. (B) A representative *in vivo* HT1080 EB3-mApple cell, where MT tracks are pseudo-colored according to the indicated feature above each image. (C) Illustrative tracks showing varying levels of MT Orientation and Coherence (tracks are colored according to cellular coherence). Tracks were quantified for a total of n=73 *in vivo* and *in vitro* cells.

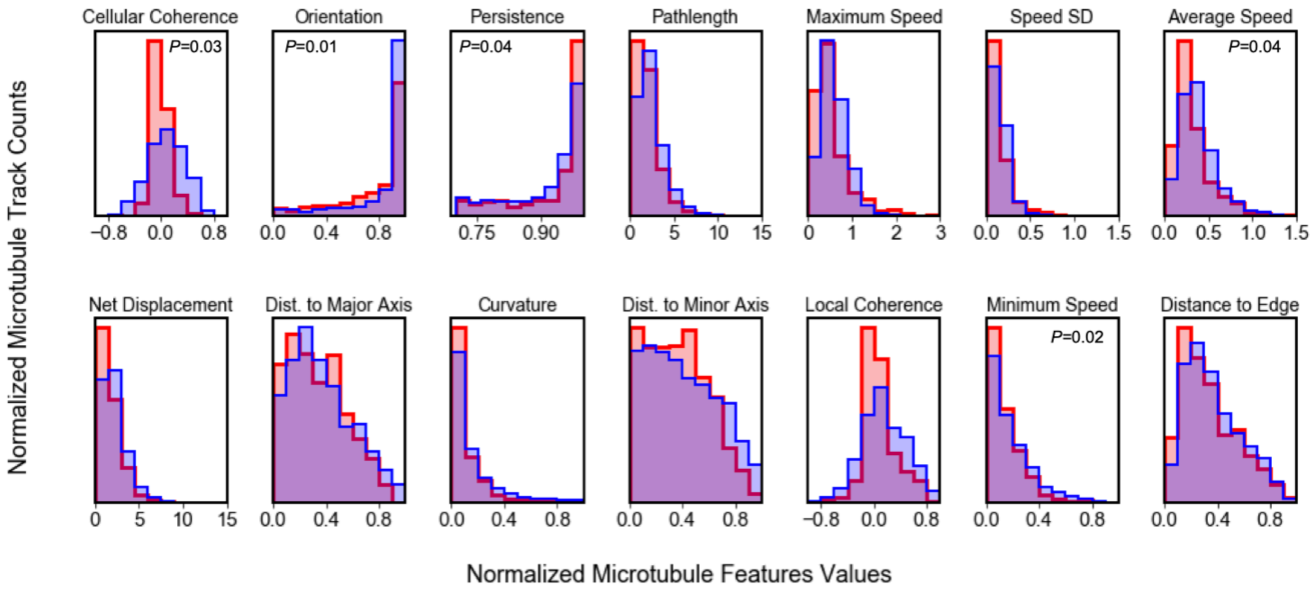

Supplementary Figure 4. Quantifying MT dynamics in ES2 xenografts. Distributions of MT dynamics imaged in ES2-EB3-mApple cells imaged *in vitro* or within ~2 week old subcutaneous xenografts in the dorsal window chamber model of nu/nu hosts (n=2,857 total tracks across 42 total cells and 5 tumors). *Two-tailed permutation test was performed for each distribution.

two-way ANOVA table (*P*-values)

Dependent Variable:	MT coherence	MT orientation	MT coherence	MT orientation
Independent variable: *in vivo* vs *in vitro*	0.014	0.036	0.004	0.006
Independent variable: Cell shape	0.72	0.057	0.63	0.035
	cell circularity		cell eccentricity	

Supplementary Figure 5. Measuring MT dynamics independent of cell shape. Statistical significance between conditions (row interactions) and cell shape (column interactions) were calculated using a two way ANOVA (n=69 cells). ANOVA interaction terms between the independent variables were not significant.

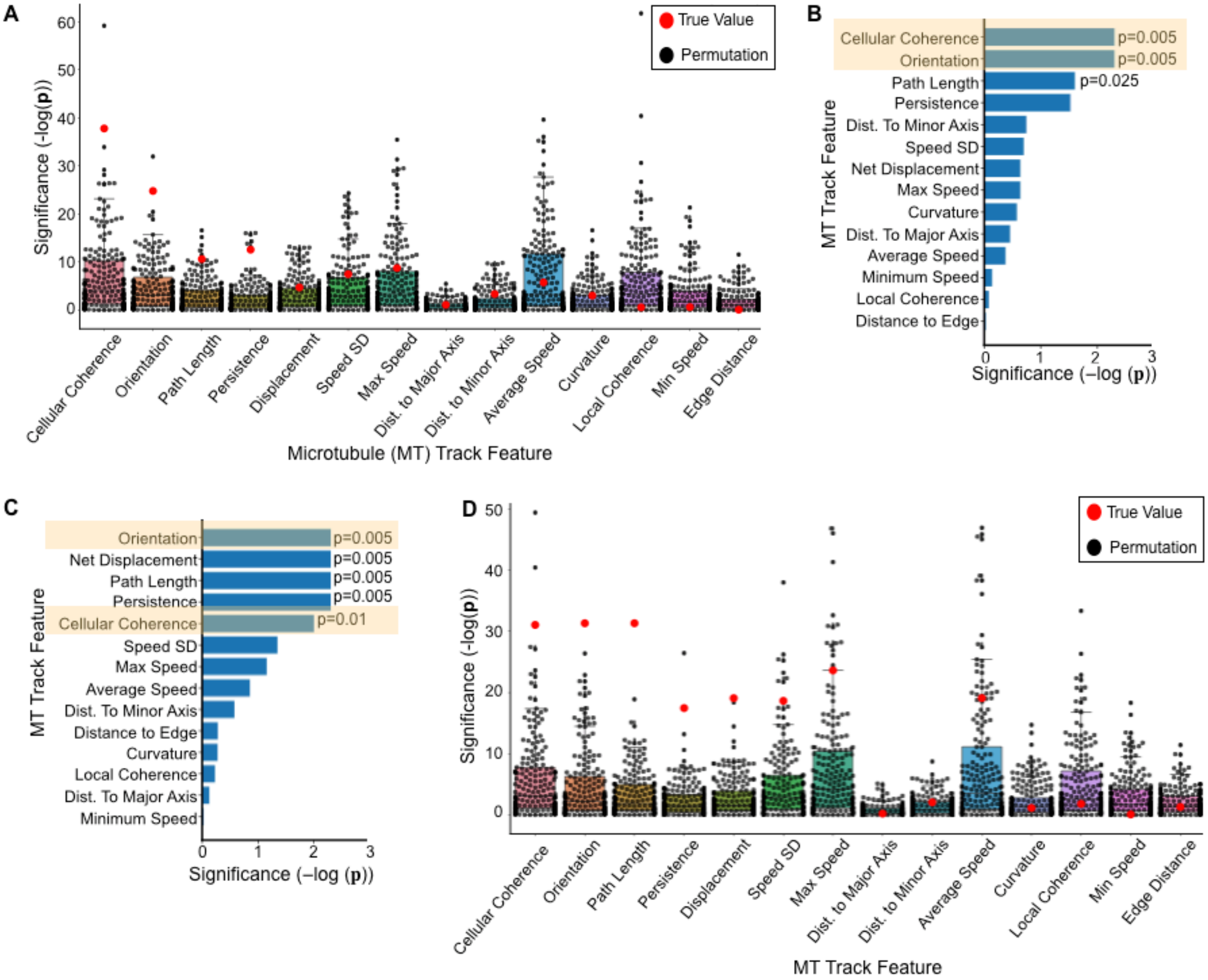

Supplementary Figure 6. Permutation testing of MT track statistics.
(A) A two-tailed wilcoxon rank-sum based permutation test was used to determine the statistical significance for each of the 14 MT track features between *in vivo* and *in vitro* HT1080 EB3-mApple cell populations. The wilcoxon statistic for the permutation (black) was compared to the wilcoxon statistic comparing the true *in vivo* and *in vitro* MT track distributions (red). (B) Corrected p-values were derived from the permutation test results shown in A (For A and B, n= 4983 tracks across 58 cells; 200 permutations). (C) To ensure that significance was not due to presence of incompletely imaged cells, the wilcoxon rank-sum permutation test was repeated after removing cells that were less than 80% visible. (D) Corresponding to C, the permutations (black) and true two-tailed wilcoxon test statistic (red) comparing *in vivo* and *in vitro* HT1080 EB3-mApple included cells mostly in the field of view (For C and D, n= 4673 tracks across 58 cells; 200 permutations). Additional un-occluded cells were added to the analysis to ensure that the number of cells remains constant. P-values for B and C were computed using a two-tailed permutation test. For A and D, a swarm plot overlays the box plot. The bars of the box plot represent 1.5*IQR-Q1, Q1/25th percentile, median, 75th percentile, 1.5IQR*Q3, and whiskers represent data points falling outside this range.

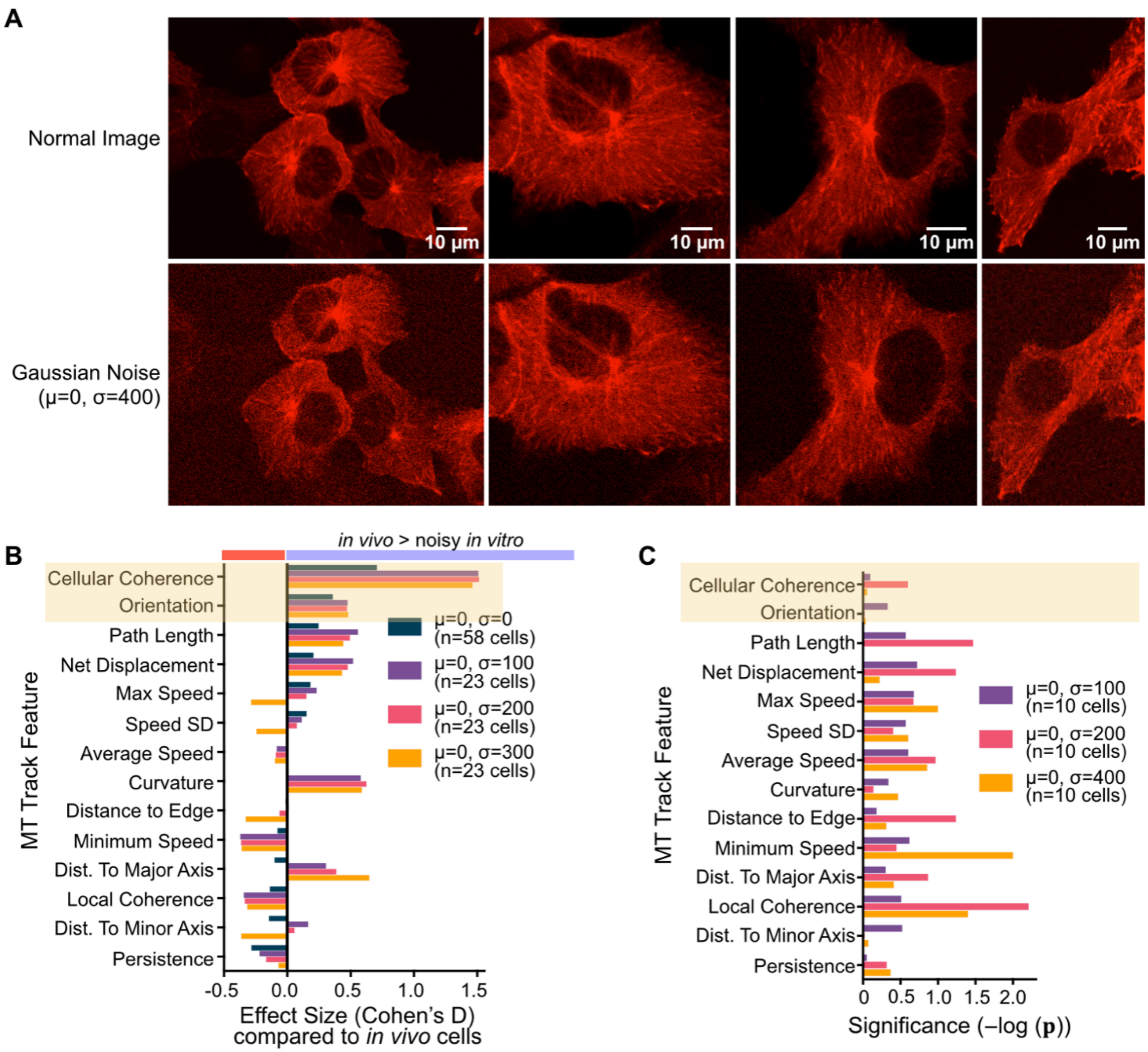

Supplementary Figure 7. The effect of artificial image noise on MT track statistics.
(A) Various levels of gaussian noise (μ=0, σ=100, 200, and 400 pixels) were added to images of HT1080 EB3-mApple cells grown in 2D culture. (B) The effect size between MT tracks from noisy *in vitro* images shown in A and HT1080 EB3-mApple *in vivo* images was measured using Cohen's D statistic (n=23 cells). These data show that relatively greater MT cellular coherence and orientation seen *in vivo* are robust to added noise in the *in vitro* images. (C) 14 MT track features were statistically compared between noisy *in vitro* images and the original, clean (μ=0, σ=0) *in vitro* images (2-tailed permutation test, n=10 cells), again suggesting that MT cellular coherence and orientation observations are robust to added noise in the imaging.

	Tracking Parameters (Threshold, Max Gap, Minimum Length)								P-Value
	4,2,4	4,4,3	3,0,4	3,2,4	3,3,4	5,4,3	4,0,3	5,0,3	
Orientation	0.68	0.67	0.69	0.68	0.68	0.66	0.67	0.66	1
Distance to Edge	269.09	261.74	267.27	271.08	269.66	248.70	261.74	250.99	0.97
Curvature	4.40	3.41	6.81	6.98	6.92	3.29	3.41	3.21	0.09
Distance to Minor Axis	0.45	0.45	0.46	0.46	0.46	0.46	0.45	0.45	1
Distance to Major Axis	0.40	0.40	0.42	0.42	0.42	0.40	0.40	0.40	1
Displacement (µm)	2.42	2.29	2.48	2.46	2.46	2.15	2.29	2.14	0.01*
Path Length (µm)	2.57	2.40	2.63	2.61	2.61	2.26	2.40	2.25	0.01*
Persistence	0.95	0.96	0.95	0.95	0.95	0.96	0.96	0.96	0.01*
Average Speed (µm/s)	0.31	0.37	0.33	0.33	0.33	0.34	0.37	0.34	0.97
Max Speed (µm/s)	0.52	0.58	0.55	0.54	0.55	0.55	0.58	0.55	1
Min Speed (µm/s)	0.15	0.20	0.16	0.16	0.16	0.17	0.20	0.17	0.68
Speed SD	0.13	0.14	0.14	0.14	0.14	0.14	0.14	0.14	0.99
Local Coherence	0.39	0.32	0.36	0.35	0.35	0.33	0.32	0.33	0.95
Cellular Coherence	0.22	0.17	0.17	0.17	0.16	0.19	0.17	0.19	0.97

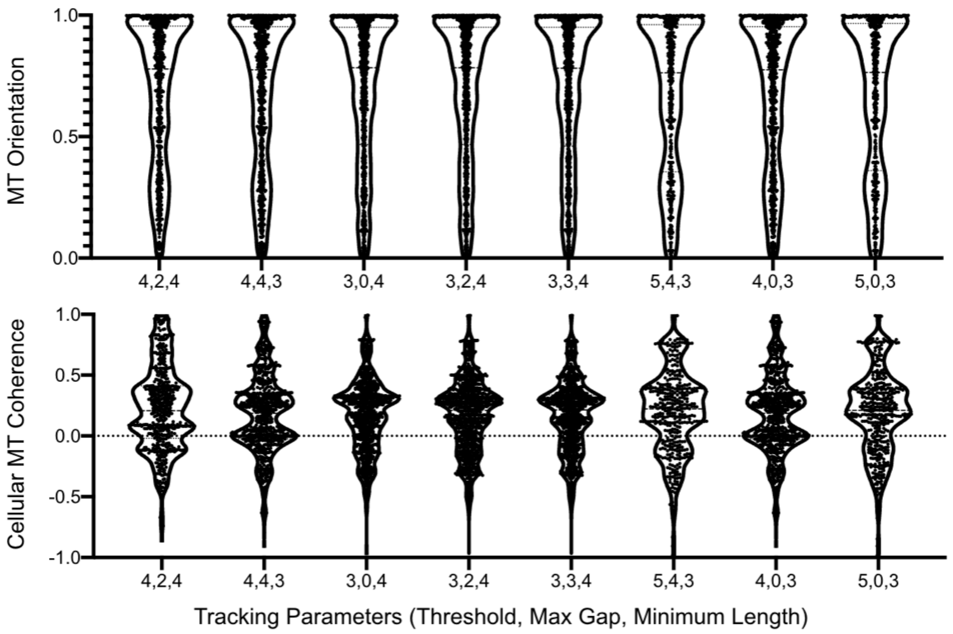

Supplementary Figure 8. Parameter sensitivity analysis in MT tracking. PlusTipTracker algorithm parameters were varied and MT track features were recomputed using *in vitro* HT1080-EB3-mApple cells, and single-track distributions are shown at bottom. Displacement, persistence, and track length but not orientation or cellular MT coherence were sensitive to the parameter changes (*kruskal-wallis (one-sided) based permutation test; n=15 cells; center bar represent the median).

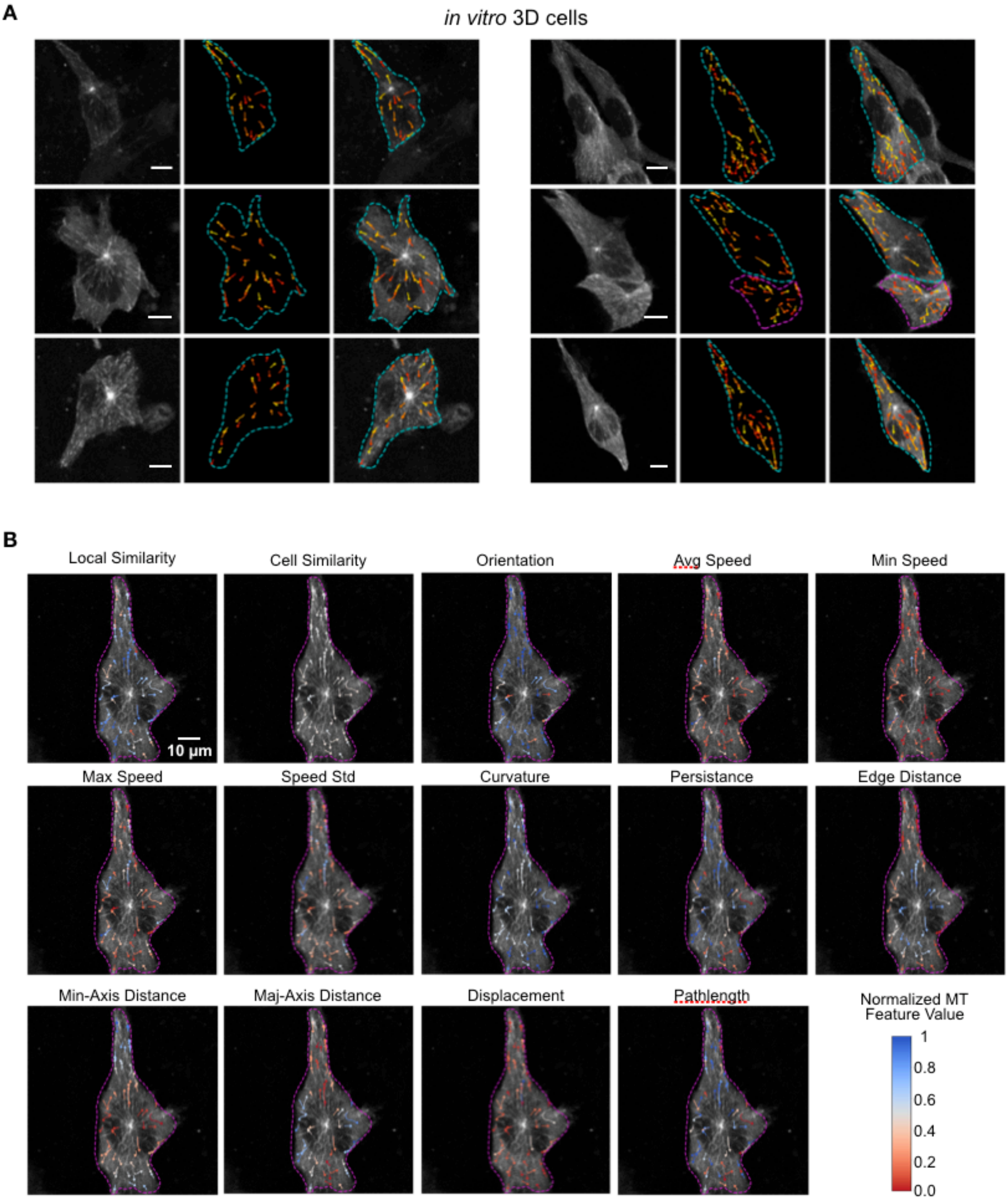

Supplementary Figure 9. Representative imaging of tumor cells in 3D culture.
(A) Representative confocal images of HT1080 EB3-mApple cells grown in 3D collagen I gel culture with computationally-identified MT tracks randomly colored (scale bar=10μm). (B) A representative HT1080 EB3-mApple cell grown in 3D collagen I gel culture pseudo-colored according to the indicated MT feature above each image. A total of n=1325 tracks across n=12 *in vitro* 3D cells were analyzed.

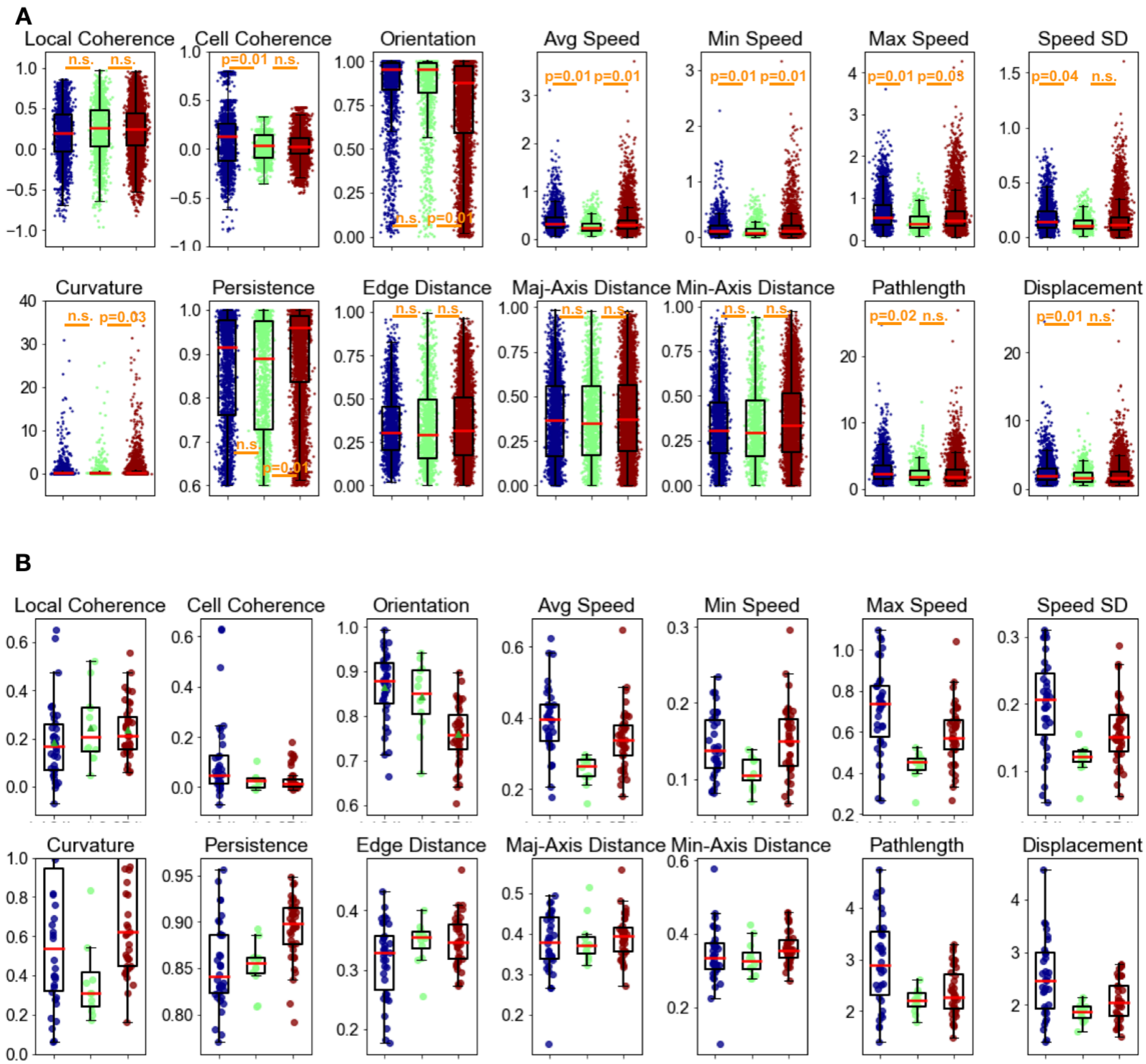

Supplementary Figure 10. Individual track and cell-average distributions of MT features. (A) Box plots show all MT tracks for each of the 14 track features. Each point corresponds to an individual MT track from an HT1080 EB3-mApple cell *in vitro* (red), *in vitro* 3D (green), or *in vivo* (blue) (total n=9,451 tracks; *two-tailed permutation test). The red line denotes the median of all MT tracks under the indicated condition. (B) Box plots show the cell medians for each MT track feature. HT1080 EB3-mApple cells were either grown on 2D culture (*in vitro*, red), grown in 3D collagen I gel (*in vitro* 3D, green), or grown *in vivo* (blue). The red bar denotes the median of the cell medians (total n=85 cells). For all, box plot defined as Q1/25%tile, median, Q3/75%tile with outliers falling outside Q3/Q1±1.5*IQR.

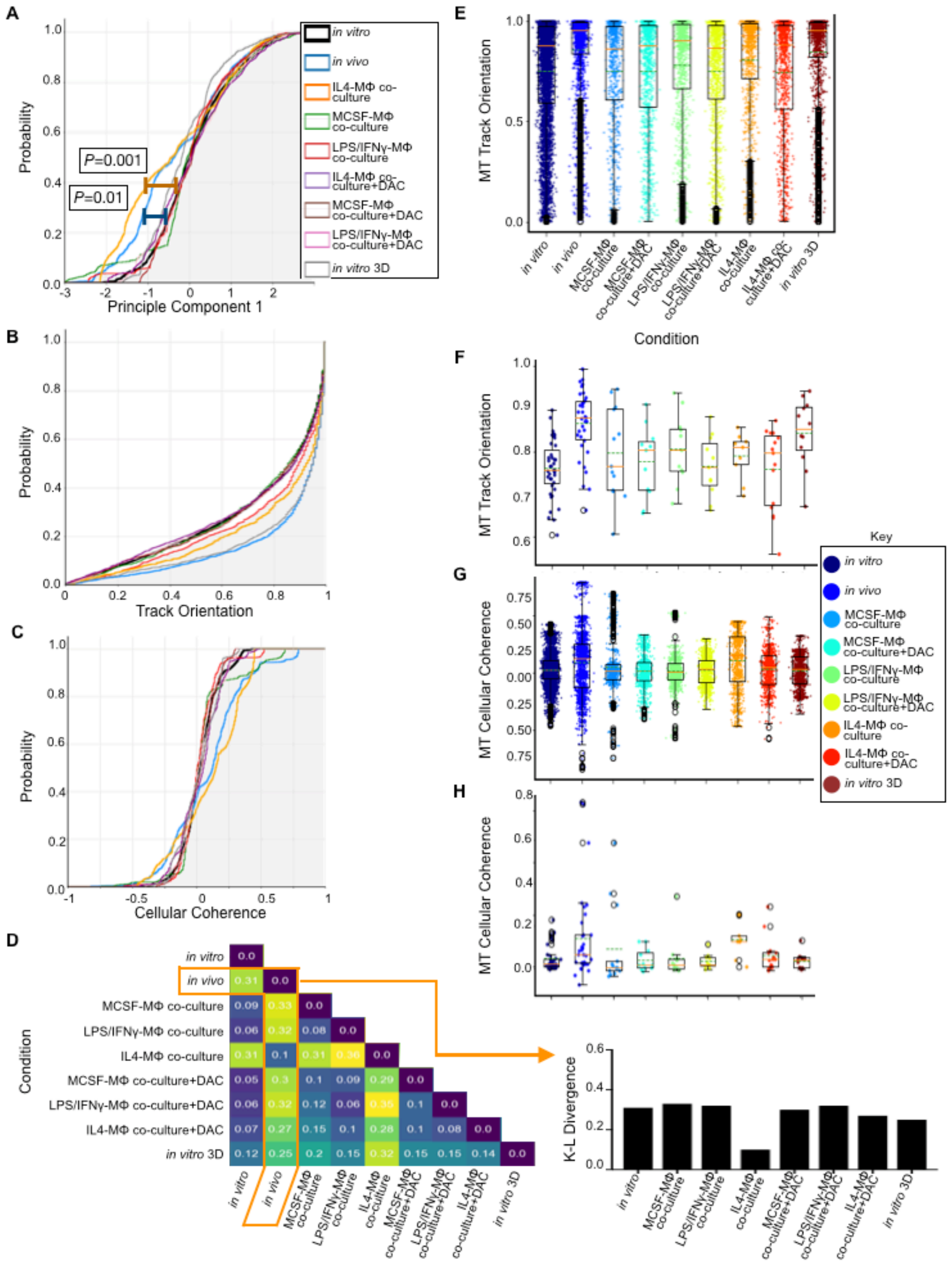

Supplementary Figure 11. Quantitative comparison of MT behavior under distinct culture conditions. (A) Covarying MT cellular coherence and orientation values were combined into a principal component for each MT track, and the cumulative probability distribution of principal component scores (PC scores) was then calculated for each culture condition (*two-tailed permutation test), reproduced for reference from Fig. 4C. Corresponding to A, the cumulative probability distribution of (B) MT Orientation values and (C) MT Cellular Coherence values were separately calculated for each culture condition (D). From the PC scores, the K-L divergence was computed between distributions from each culture condition. The bar-plot compares the K-L divergences of the *in vivo* PC score distribution to PC score distributions under all other conditions. (E-H) Under each culture condition, the orientation value of (E) all tracks and the (F) cell-averages were calculated and visualized. Likewise the cellular coherence value for (G) all tracks and the (H) cell averages are shown. The red line denotes the median value of either all tracks(E, G) or the cell averages (F, H). For all (A-H), n=15,965 tracks from n=155 total cells. All box plots are defined as Q1/25%tile, median, Q3/75%tile with outliers falling outside Q3/Q1±1.5*IQR.

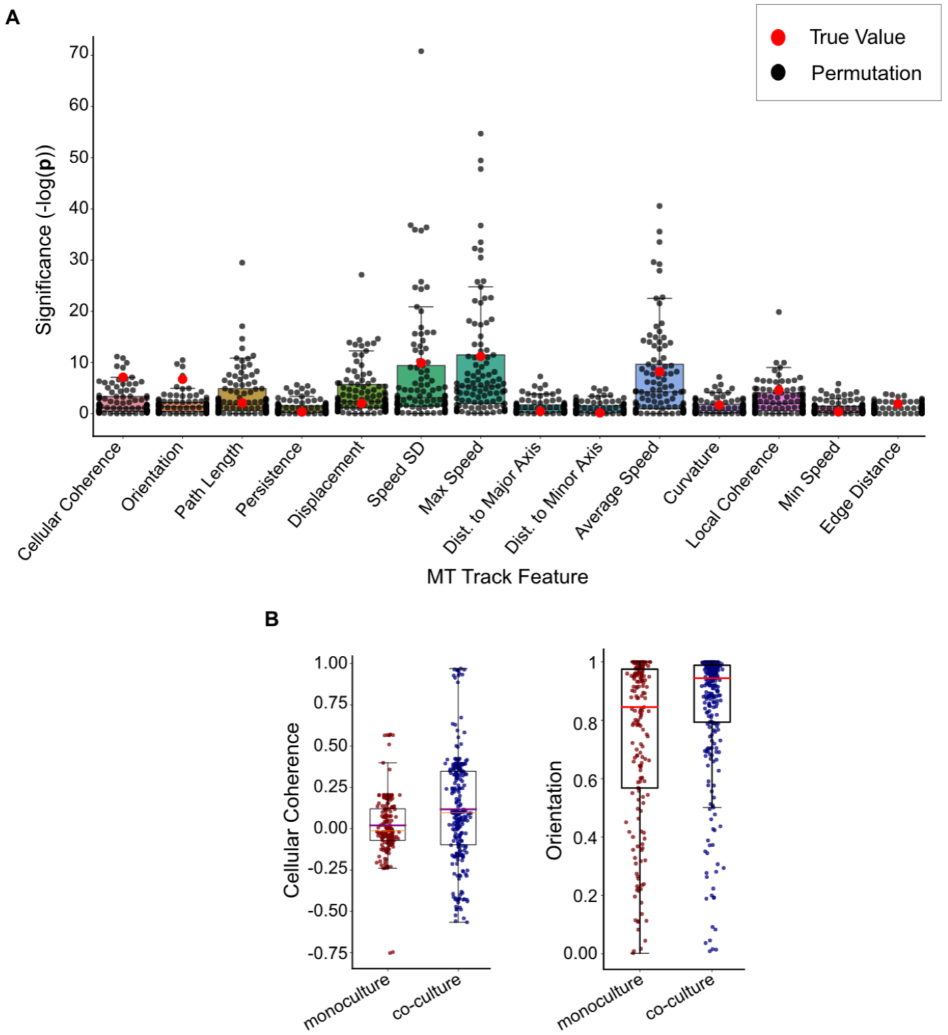

Supplementary Figure 12. Permutation statistics and individual track-level data in the ES2 co-culture model.

(A) A wilcoxon rank-sum based permutation test was used to determine the statistical significance for each of the 14 MT track features between ES2 EB3-mApple cells cultured alone (monoculture) or cultured with IL4-MΦ (co-cultured). The wilcoxon statistic for the permutation (black) was compared to the wilcoxon statistic comparing the true mono-culture and co-culture MT track distributions (red). (B) Box plots show the distribution of MT track orientation and cellular coherence features. Each point overlaid on the box plot corresponds to an individual MT track from an ES2 EB3-mApple cell grown in monoculture (red) or in co-culture with IL4-MΦ (blue)(red line denotes median). (A-B) N=1,424 tracks across n=33 total ES2 cells were analyzed. All box plots are defined as Q1/25%tile, median, Q3/75%tile with outliers falling outside Q3/Q1±1.5*IQR.

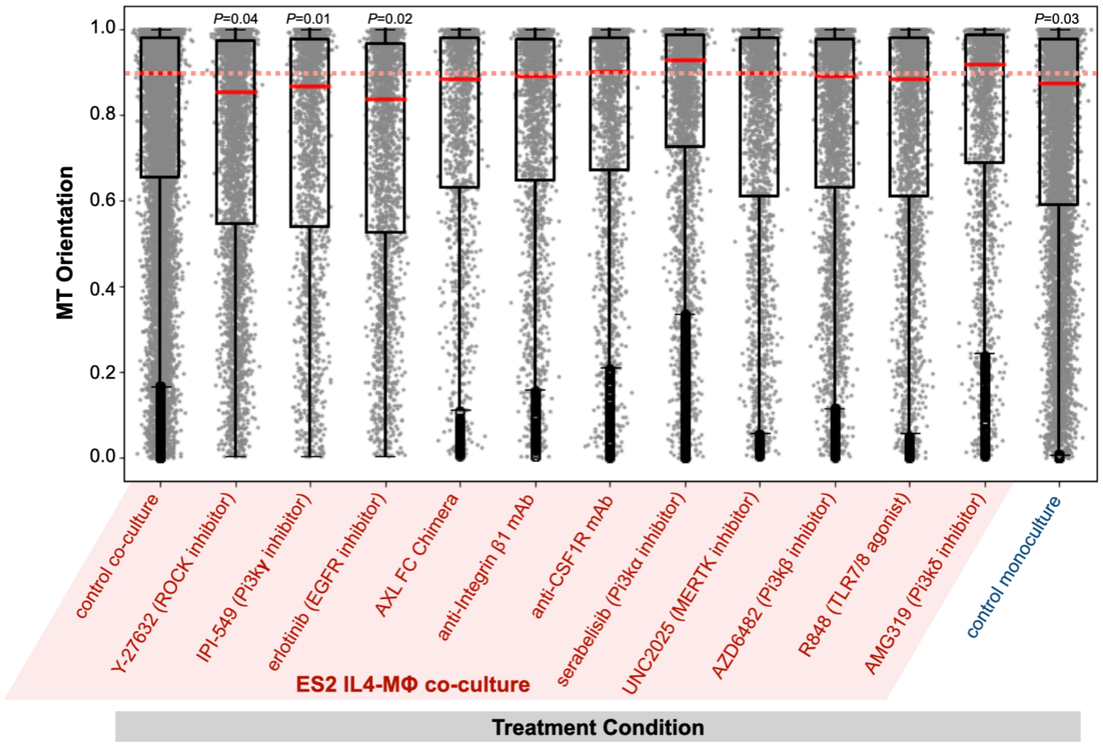

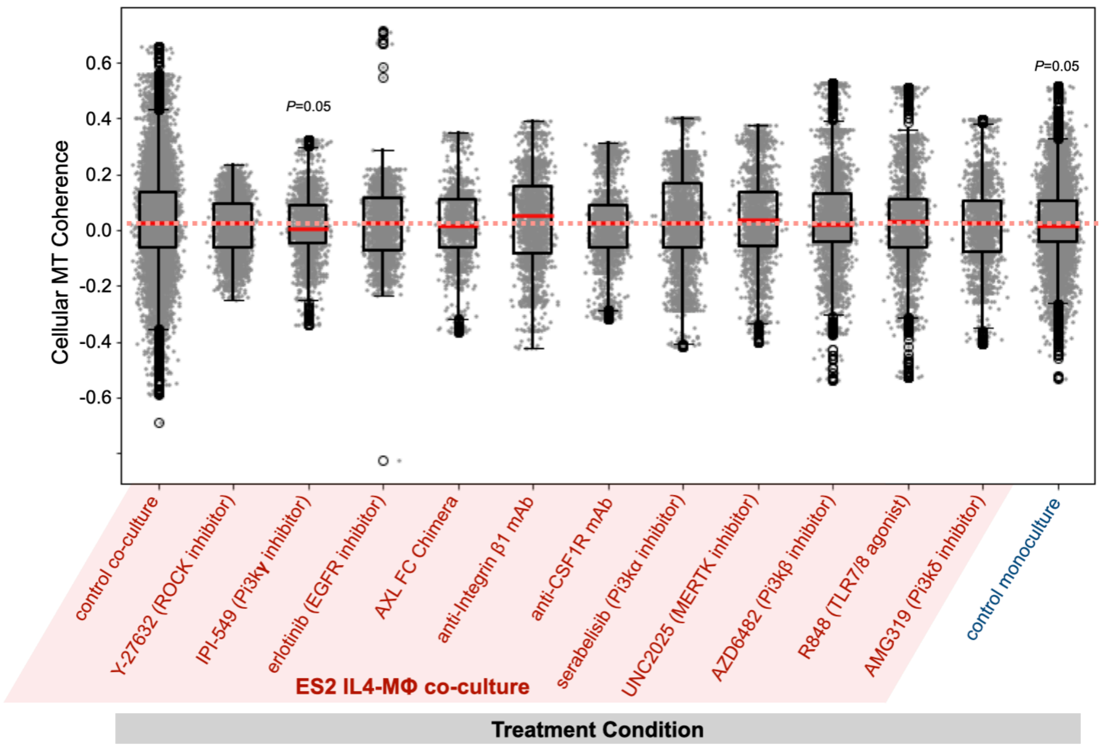

Supplementary Figure 13. ES2 MT dynamics in response to targeted reagents and drugs. Corresponding to Fig. 5c, MT orientation and coherence were measured following treatment with the indicated targeted compounds in ES2-EB3-mApple cells, shown as single-track distributions (*two-tailed permutation test comparing treatment with untreated co-culture; n=10,968 tracks from n=118 total cells). Box plot bars represent the minimum, 25%tile, median, 75%tile, and maximum values.

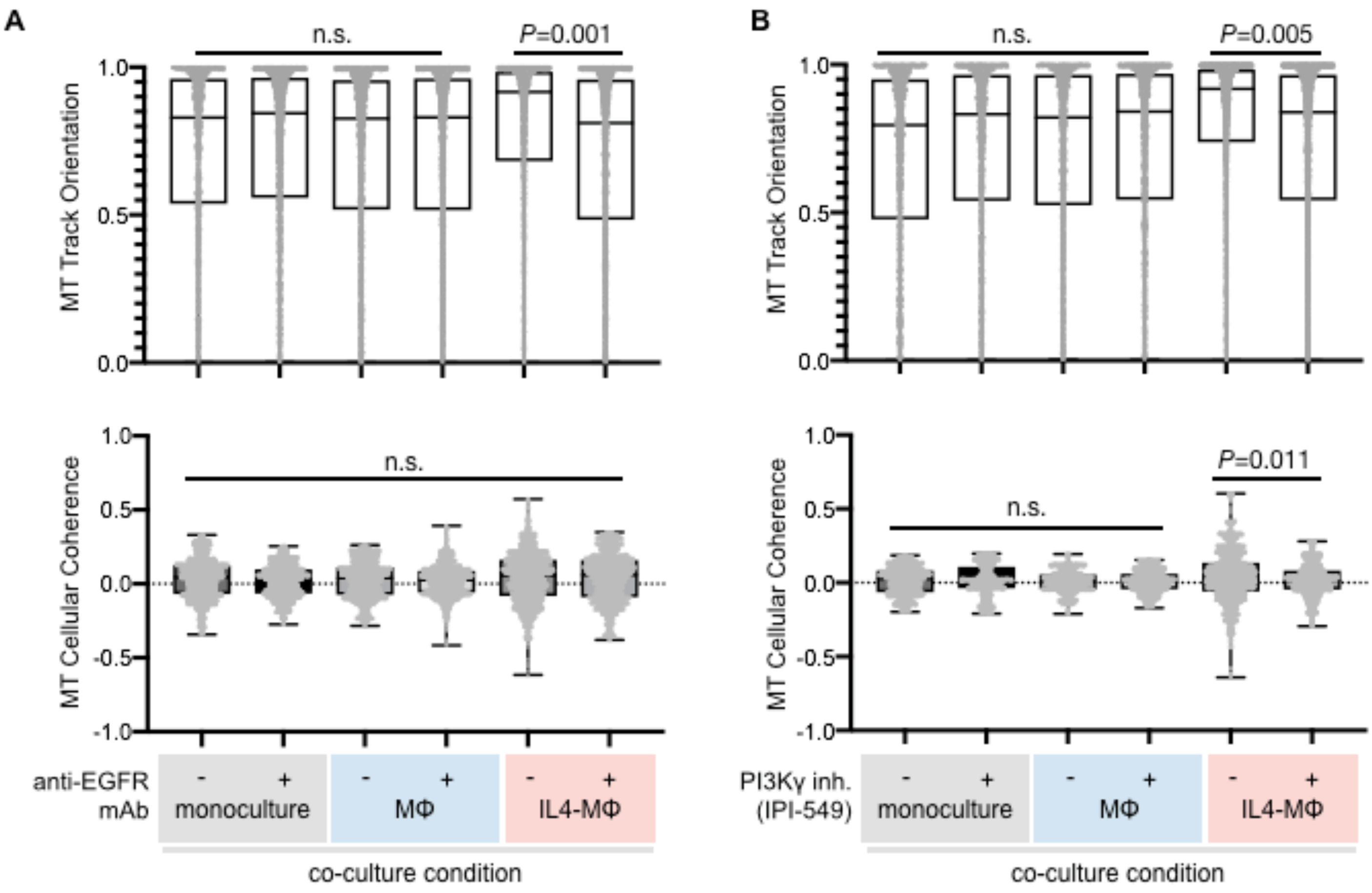

Supplementary Figure 14. HT1080 MT dynamics in response to targeted reagents and drugs. Corresponding to Fig. 5d and Fig. 9, single-track distributions are shown for HT1080-EB3-mApple cells following treatment with anti-EGFR antibody (A: n=13,380 tracks from n=80 total cells) and IPI-549 (B: n=16,427 tracks from n=112 total cells). Box plot bars represent the minimum, 25%tile, median, 75%tile, and maximum values. Significance values were computed using a two-tailed permutation test.

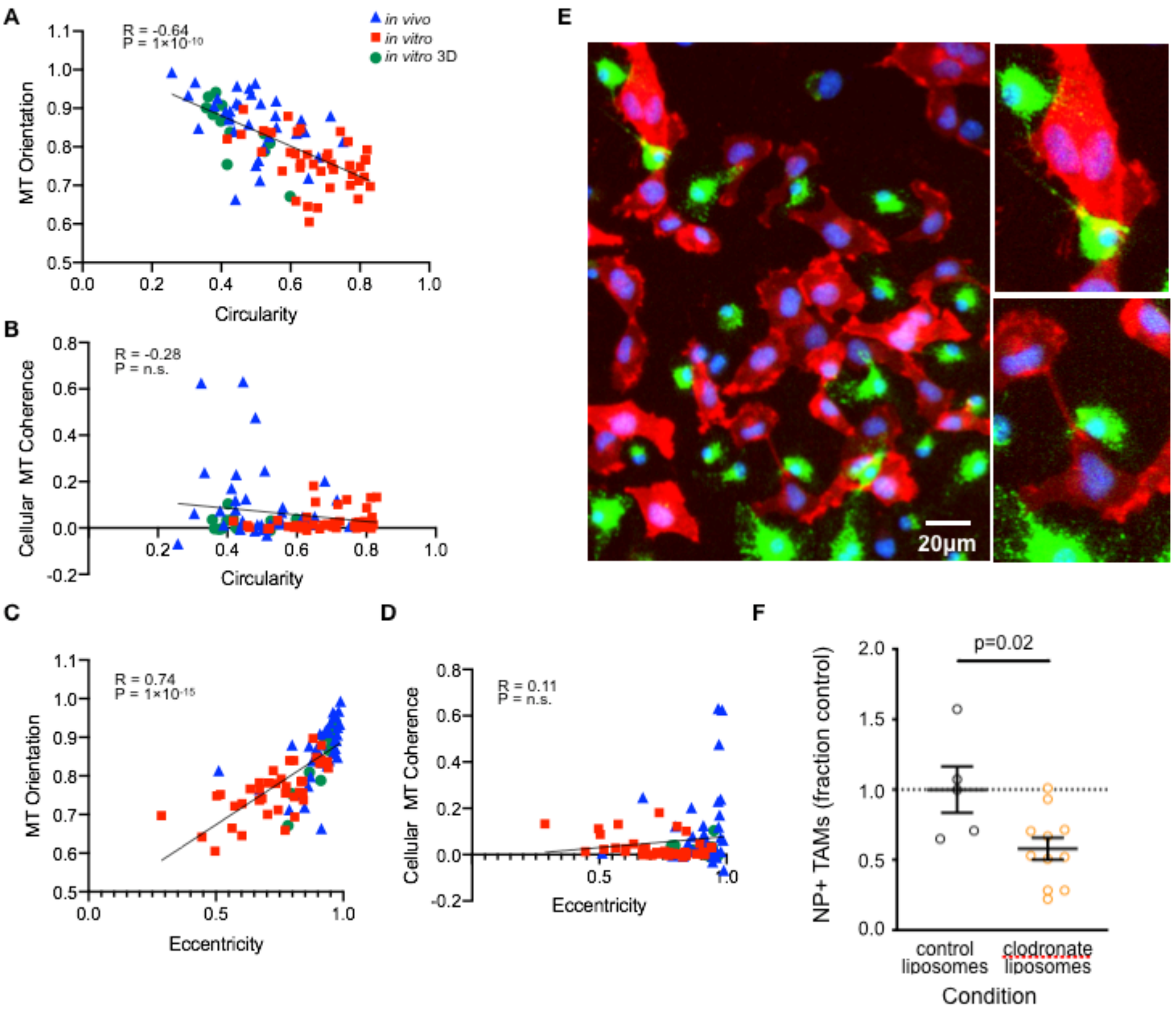

Supplementary Figure 15. Correlations between cell shape and MT behaviors.
(A) Cellular circularity was correlated with average MT orientation across individual HT1080 EB3-mApple cells grown under the indicated conditions (data points denote individual cells; *two-tailed exact test). (B-D) Similar to A, (B) cellular circularity was correlated to MT cellular coherence, cellular eccentricity was correlated to (C) MT orientation and (D) MT cellular coherence across individual HT1080 cells (A-D: *two-tailed F-test; n=85 cells from three different experimental conditions). (E) Representative images of HT1080-mem-mApple cells in co-culture with NP-labeled IL4-MΦ, highlighting instances of co-localized MΦ and tumor cell protrusion. (F) Quantification of NP+ cells to determine relative MΦ content present in intraperitoneal ES2-mClover tumors (two tailed unpaired t-test; n=16 tumors; error bars denote mean±s.e.m.). Signficance tests used in linear regression analysis(A-D) measure if slope is significantly nonzero.

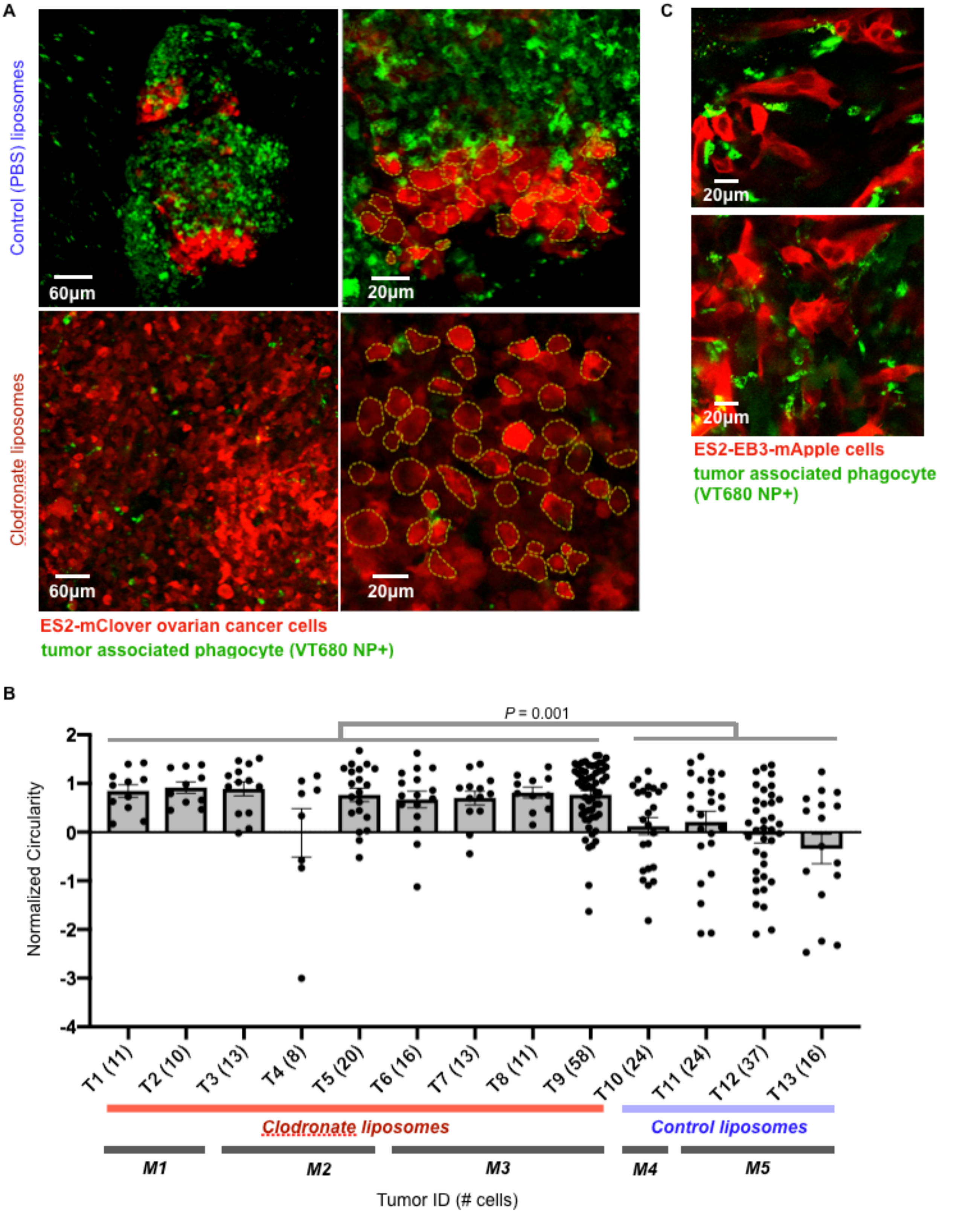

Supplementary Figure 16. Quantifying tumor cell shape in response to clodronate liposome treatment. Corresponding to Fig. 6h, ES2 tumors via intraperitoneal injectection were treated with liposomes containing either clodonate or PBS as a vehicle control, and imaged confocally for cell shape. (A) Representative images and (B) quantification of single-cell circularities across tumors within the cohorts are shown (*two-tailed t-test, n=261 cells across n=13 tumors; error bars denote mean±s.e.m. for each group). (C) ES2 tumor cells were also implanted subcutaneously and imaged using a dorsal window chamber.

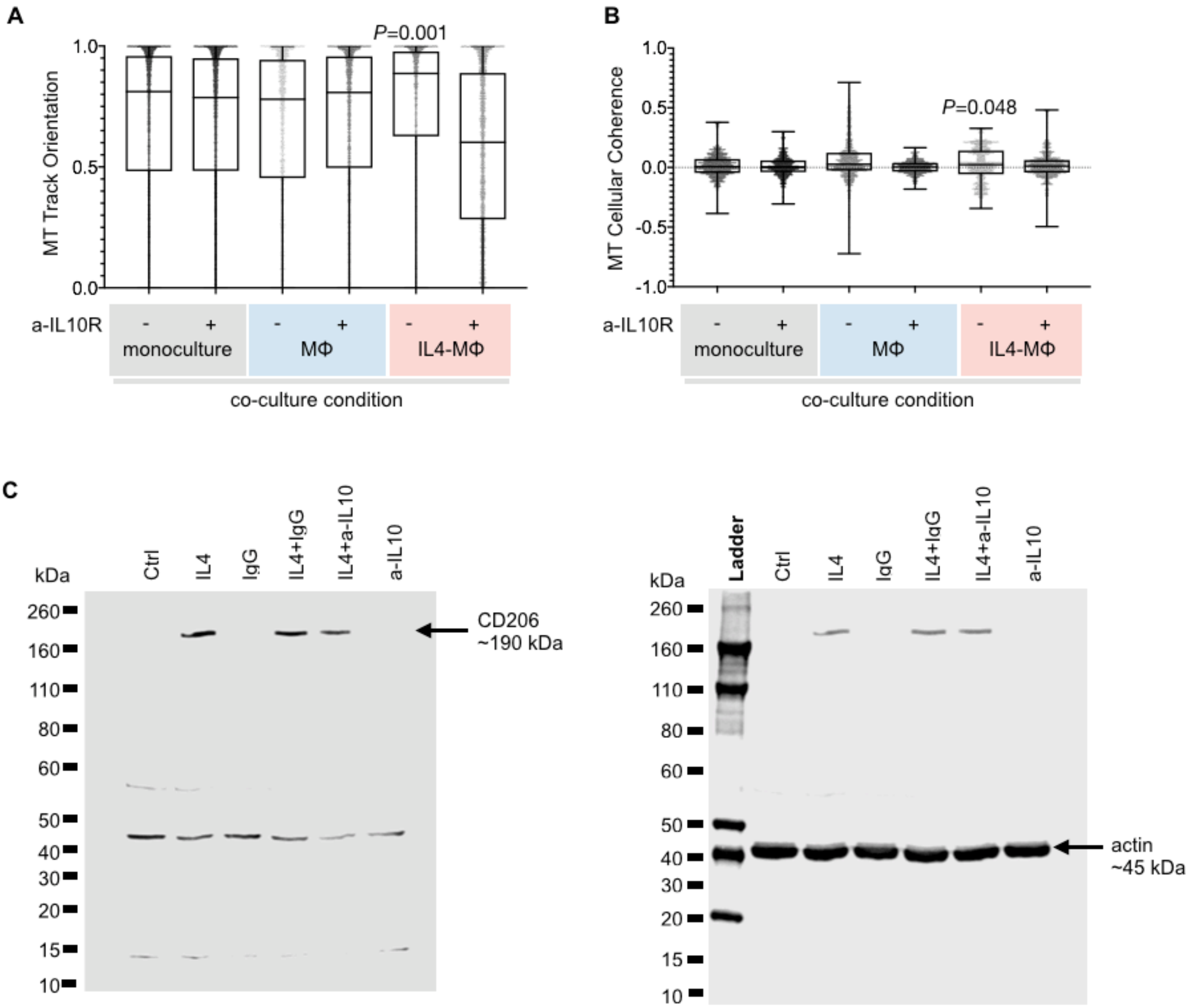

Supplementary Figure 17. The effect of Anti-IL10R antibody treatment on MT dynamics. MT orientation (A) and coherence (B) of HT1080-EB3-mApple cells were measured following treatment with anti-IL10R antibody (A-B: n=21,218 tracks from 99 total cells). Significance values were computed by a two tailed permutation comparing two groups: IL4-MΦ co-culture vs all other cells. Box plot bars represent the minimum, 25%tile, median, 75%tile, and maximum values. (C) Full image of blot corresponding to fig. 9b. The blot was probed first with rabbit anti-CD206 primary antibody, followed by anti-rabbit secondary antibody (left). The blot was washed and then probed with mouse anti-actin primary antibody, followed by anti-mouse secondary antibody (right). CD206 band in the blot on the right is the result of residual HRP activity from the first blot.

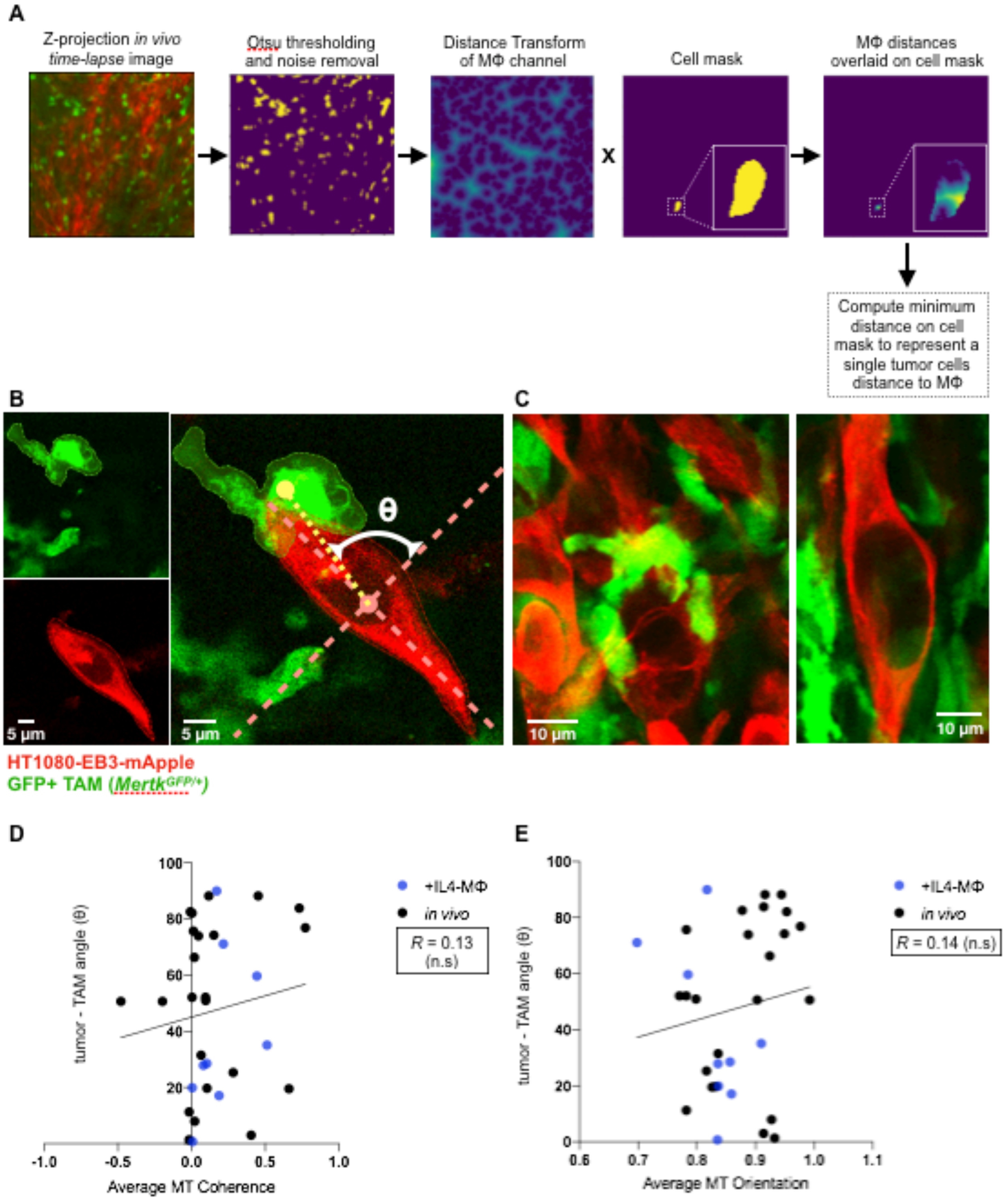

Supplementary Figure 18. Quantifying macrophage proximity and subcellular orientation to tumor cells. (A) Image processing pipeline for how the distance of nearest macrophage was calculated for HT1080 cells (corresponding to Fig. 7f). (B) Example of tumor cell near a TAM located at its major length axis, for which quantification of "tumor-macrophage angle" is possible, in this case leading to a cosine value near 1.0. (C) Such "tumor-macrophage angles" cannot be calculated from cases where no single TAM can be assigned as nearest to the tumor cell, and therefore were excluded from analysis of "tumor-macrophage angles." (D-E) Cell-by-cell quantification of cellular MT coherence (D) and orientation (E) relative to the measured tumor-macrophage angle (D-E: *two-tailed F-test; n=32 cells).
