# Supplementary material for: In vivo microscopy reveals macrophage polarization locally promotes coherent microtubule dynamics in migrating cancer cells
Source: Nat Commun. 2020 Jul 14;11:3521. doi: 10.1038/s41467-020-17147-y (PMC7360550; doi:10.1038/s41467-020-17147-y)
Supplement: Supplementary file 2 — Description of Additional Supplementary Files [file 41467_2020_17147_MOESM2_ESM.pdf]

Description of Additional Supplementary Files

File Name: Supplementary Movie 1
Description: Intravital microscopy and computational tracking of MT tracks in HT1080-EB3-mApple xenograft tumors.

File Name: Supplementary Movie 2
Description: Intravital microscopy and computational tracking of MT tracks in HT1080-EB3-mApple xenograft tumor treated with aIL10R and corresponds to Fig. 9.
