# Supplementary material for: In vivo microscopy reveals macrophage polarization locally promotes coherent microtubule dynamics in migrating cancer cells
Source: Nat Commun. 2020 Jul 14;11:3521. doi: 10.1038/s41467-020-17147-y (PMC7360550; doi:10.1038/s41467-020-17147-y)
Supplement: Supplementary file 5 — Reporting Summary [file 41467_2020_17147_MOESM5_ESM.pdf]

nature research

Corresponding author(s): Miles Miller, Ralph Weissleder

Last updated by author(s): May 20, 2020

Reporting Summary

Nature Research wishes to improve the reproducibility of the work that we publish. This form provides structure for consistency and transparency in reporting. For further information on Nature Research policies, see Authors & Referees and the Editorial Policy Checklist .

Statistics

For all statistical analyses, confirm that the following items are present in the figure legend, table legend, main text, or Methods section.

n/a	Confirmed	
☐	☒	The exact sample size (*n*) for each experimental group/condition, given as a discrete number and unit of measurement
☐	☒	A statement on whether measurements were taken from distinct samples or whether the same sample was measured repeatedly
☐	☒	The statistical test(s) used AND whether they are one- or two-sided<br>*Only common tests should be described solely by name; describe more complex techniques in the Methods section.*
☐	☒	A description of all covariates tested
☐	☒	A description of any assumptions or corrections, such as tests of normality and adjustment for multiple comparisons
☐	☒	A full description of the statistical parameters including central tendency (e.g. means) or other basic estimates (e.g. regression coefficient) AND variation (e.g. standard deviation) or associated estimates of uncertainty (e.g. confidence intervals)
☐	☒	For null hypothesis testing, the test statistic (e.g. *F*, *t*, *r*) with confidence intervals, effect sizes, degrees of freedom and *P* value noted<br>*Give P values as exact values whenever suitable.*
☒	☐	For Bayesian analysis, information on the choice of priors and Markov chain Monte Carlo settings
☒	☐	For hierarchical and complex designs, identification of the appropriate level for tests and full reporting of outcomes
☐	☒	Estimates of effect sizes (e.g. Cohen's *d*, Pearson's *r*), indicating how they were calculated

Our web collection on statistics for biologists contains articles on many of the points above.

Software and code

Policy information about availability of computer code

Data collection	Imaging data was obtained via intravital or normal confocal microscopy performed by an Olympus FV1000 multiphoton imaging system. Following imaging, microtubule tracks were obtained using the UTrack software developed by the Danuser Group at UT Southwestern.
Data analysis	Data analysis was conducted in Prism 8 , SPRING (accessed Dec. 2019 - Jan 2020), python3.6 (numpy, matplotlib packages), and MATLAB_R2016b Github link to the MT track feature extraction pipeline is here: https://github.com/gluthria/MT_Dynamics

For manuscripts utilizing custom algorithms or software that are central to the research but not yet described in published literature, software must be made available to editors/reviewers. We strongly encourage code deposition in a community repository (e.g. GitHub). See the Nature Research guidelines for submitting code & software for further information.

Data

Policy information about availability of data

All manuscripts must include a data availability statement. This statement should provide the following information, where applicable:

- Accession codes, unique identifiers, or web links for publicly available datasets
- A list of figures that have associated raw data
- A description of any restrictions on data availability

The source data underlying all main figures (Fig. 1d-f, 2b-e, 3c-d, 4a-d, 6a-c,e,g-h, 7a,c,f, 8a-f, 9a-f,h-i) and supplementary figures are provided as a Source Data file. All additional data that support the findings of this study are available from the authors on reasonable request. GSE accession numbers for publicly available data used in this study are GSE118828[https://www.ncbi.nlm.nih.gov/geo/query/acc.cgi?acc=GSE118828]), GSE72056[https://www.ncbi.nlm.nih.gov/geo/query/acc.cgi?acc=GSE72056]), GSE103322[https://www.ncbi.nlm.nih.gov/geo/query/acc.cgi?acc=GSE103322]). Kaplan-Meier survival curves were derived from cBioportal [https://www.cbioportal.org/] which uses data from the Cancer Genome Atlas (TCGA).

Field-specific reporting

Please select the one below that is the best fit for your research. If you are not sure, read the appropriate sections before making your selection.

- [x] Life sciences
- [ ] Behavioural & social sciences
- [ ] Ecological, evolutionary & environmental sciences

For a reference copy of the document with all sections, see nature.com/documents/nr-reporting-summary-flat.pdf

Life sciences study design

All studies must disclose on these points even when the disclosure is negative.

Sample size

Sample sizes were determined by counting the number of MT tracks, cells, tumors, or mice depending on the analysis. For track based analyses sample sizes were not predetermined and all the tracks imaged computationally determined were used in the analysis. Sample sizes for cells, tumors, or mice were chosen based on estimated effect sizes from prior studies listed below with this xenograft model and in vitro cell culture experiments.

Miller, M. A. et al. Modular Nanoparticulate Prodrug Design Enables Efficient Treatment of Solid Tumors Using Bioorthogonal Activation. ACS Nano (2018).
Pineda, J. J. et al. Site occupancy calibration of taxane pharmacology in live cells and tissues. Proc Natl Acad Sci U S A 115, E11406-E11414 (2018).
Chittajallu, D. R. et al. In vivo cell-cycle profiling in xenograft tumors by quantitative intravital microscopy. Nat Methods 12, 577-585 (2015).
Miller, M. A. et al. Predicting therapeutic nanomedicine efficacy using a companion magnetic resonance imaging nanoparticle. Sci Transl Med 7, 314ra183 (2015).
Wang, Stephanie J., et al. "Efficient blockade of locally reciprocated tumor-macrophage signaling using a TAM-avid nanotherapy." Science Advances 6.21 (2020): eaaz8521.

Data exclusions

No data was excluded in our analysis.

Replication

For all our analyses we performed experiments across replicates, all of which were included in our analyses. Number of replications were chosen to meet standard practices and aforementioned criteria as related to the sample sizes described above. All replicate numbers are explicit in the figure legends. All experiments were performed with n >= 2 independent experiments.

Randomization

To test significance robustly, we performed a permutation test which involved randomization of cell/track labels followed by comparing the naive statistical significance to the statistical significance of each permutation. The permutation was conducting between different experimental groups. Groups were defined by treatment. Therefore, samples that undergo different treatment (drug treatment, co-culture conditions, M-phage polarization, etc.) were put in different groups. Animal assignment into treatment groups, as with the aIL10R experiment, was performed randomly.

Blinding

Analysis was conducted via unbiased computational scripts. Therefore, the analysis is blinded to which group (treatment or control) the sample originated from.

Reporting for specific materials, systems and methods

We require information from authors about some types of materials, experimental systems and methods used in many studies. Here, indicate whether each material, system or method listed is relevant to your study. If you are not sure if a list item applies to your research, read the appropriate section before selecting a response.

Materials & experimental systems

n/a	Involved in the study	
[ ]	[x]	Antibodies
[ ]	[x]	Eukaryotic cell lines
[x]	[ ]	Palaeontology
[ ]	[x]	Animals and other organisms
[x]	[ ]	Human research participants
[x]	[ ]	Clinical data

Methods

n/a	Involved in the study	
[x]	[ ]	ChIP-seq
[x]	[ ]	Flow cytometry
[x]	[ ]	MRI-based neuroimaging

Antibodies

Antibodies used

anti-IL-10R (BioXcell; Clone # 1B1.3A, Cat #BE0050)
anti-human EGFR (anti-EGFR; BioXcell; Clone #225 Cat #BE0278)
rat IgG1 isotype control (IgG ctrl; BioXcell; Clone HRPN Cat #BP0088)
mouse IgG1 isotype control (IgG ctrl; BioXcell; Clone MOPC-21 Cat #BE0083)

	human Integrin β1/CD29 Antibody (R&D Systems; Clone # P5D2 Cat #MAB17781-SP)<br>anti-CSF1R (BioXCell; Clone # AFS98 Cat#BE0213)<br>Axl Fc Chimera (R&D Systems; Cat# 154-AL-100)<br>rabbit anti-CD206 antibody (Abcam, polyclonal Cat #ab64693)<br>mouse anti-β actin antibody (Cell Signaling Technology, clone#8H10D10, Cat #3700S)
Validation	Information regarding antibody validation are available from the manufacturer and manufacturer-provided publications at the following URLs:<br>1) https://bxcell.com/product/m-il-10r/ "The 1B1.3A antibody is a neutralizing antibody and has been shown to block the binding of human IL-10, which cross-reacts with the mouse IL-10R. However, this clone does not recognize the human IL-10R."; "Purity: >95% Determined by SDS-PAGE"<br>2) https://bxcell.com/product/invivomab-anti-human-egfr-528/ "The 528 antibody has been reported to block EGF binding to its receptor and inhibit A431 tumor formation in nude mice."; "Purity: >95% Determined by SDS-PAGE"<br>3) https://bxcell.com/product/rat-igg1-isotype-control/ "The HRPN monoclonal antibody reacts with horseradish peroxidase (HRP). Because HRP is not expressed by mammals this antibody is ideal for use as an isotype-matched control for rat IgG1 antibodies in most in vivo and in vitro applications."; "Purity: >95% Determined by SDS-PAGE"<br>4) https://bxcell.com/product/mouse-igg1-isotype-control/ "The MOPC-21 monoclonal antibody is ideal for use as a non-reactive isotype-matched control for mouse IgG1 antibodies in most in vivo and in vitro applications."; "Purity: >95% Determined by SDS-PAGE"<br>5) https://www.rndsystems.com/products/human-integrin-beta1-cd29-antibody-p5d2_mab17781 "Integrin β1/CD29 was detected in immersion fixed human peripheral blood mononuclear cells (PBMCs) using Mouse Anti-Human Integrin β1/CD29 Monoclonal Antibody (Catalog # MAB17781) at 25 µg/mL for 3 hours at room temperature."<br>6) https://bxcell.com/product/anti-cd115-anti-csf-1/ "The AFS98 antibody has been reported to deplete macrophages and block CSFR1 in vivo"; "Purity: >95% Determined by SDS-PAGE"<br>7) https://www.rndsystems.com/products/recombinant-human-axl-fc-chimera-protein-cf_154-al "Measured by its binding ability in a functional ELISA. When Recombinant Human Axl Fc Chimera is immobilized at 2 µg/mL (100 µL/well), the concentration of Recombinant Human Gas6 (Catalog # 885-GS) that produces 50% of the optimal binding response is approximately 2.5-15 ng/mL."<br>8) https://www.abcam.com/mannose-receptor-antibody-ab64693.html "Use a concentration of 1 µg/ml. Detects a band of approximately 190 kDa (predicted molecular weight: 166 kDa)."<br>9) https://www.cellsignal.com/products/primary-antibodies/b-actin-8h10d10-mouse-mab/3700 "β-Actin (8H10D10) Mouse mAb detects endogenous levels of total β-actin protein."; "Species reactivity is determined by testing in at least one approved application (e.g., western blot)."

Eukaryotic cell lines

Policy information about cell lines

Cell line source(s)	ATCC<br>Cell lines used are HT1080, ES2, and Raw264.7<br>Cell lines generated are HT1080-mem-mApple, HT1080-EB3-mApple, ES2-EB3-mApple, and ES2-mClover
Authentication	Cell lines were not further authenticated after their receipt from ATCC.
Mycoplasma contamination	Cell lines were tested for mycoplasma contamination using Lonza MycoAlert according to manufacturer's protocols. Negative test results were obtained for all cell lines used in the study.
Commonly misidentified lines (See ICLAC register)	No commonly misidentified lines were used.

Animals and other organisms

Policy information about studies involving animals; ARRIVE guidelines recommended for reporting animal research

Laboratory animals	Intravital imaging was conducted with female 4-10 week old mice of nu/nu (Cox7, MGH) and NOD.SCID MERTK GFP/+ background (the latter bred in house). Murine bone marrow-derived MΦ for in vitro co-culture studies were isolated from the femurs and tibias of female 6-8 week old C57BL/6 (JAX) and NOD.SCID MERTK GFP/+ mice.<br><br>Animals were housed in a light-dark cycle, climate (temperature and humidity via heating venting air conditioning) controlled vivarium and kept under ad libitum food and water diet supplied by the MGH Center for Comparative Medicine staff.
Wild animals	This study did not involve wild animals.
Field-collected samples	This study did not involve samples collected from the field.
Ethics oversight	Animals were used in accordance with guidelines from the Institutional Animal Care and Use Committee (IACUC) at Massachusetts General Hospital

Note that full information on the approval of the study protocol must also be provided in the manuscript.
